# Cell swelling and upright mounting-based imaging for high-resolution visualization of intracellular trafficking across the BBB using conventional confocal microscopy

**DOI:** 10.1080/10717544.2025.2608235

**Published:** 2026-01-06

**Authors:** Da Hee Oh, Ji Hee Kang, O Hyun Lee, Young Tag Ko

**Affiliations:** a College of Pharmacy and Gachon Institute of Pharmaceutical Sciences, Gachon University, Incheon, Republic of Korea

**Keywords:** Blood–brain barrier, receptor-mediated transcytosis, intracellular trafficking, confocal microscopy, direct cellular visualization, vesicle transport, cell swelling, upright mounting

## Abstract

Receptor-mediated transcytosis (RMT) represents a promising strategy for delivering macromolecular and colloidal therapeutics across the blood–brain barrier (BBB). However, mechanistic elucidation of RMT remains limited by the difficulty of visualizing subcellular trafficking pathways. Conventional imaging approaches either lack sufficient spatial resolution or require costly, technically complex instrumentation. Here, we report a cell swelling and upright mounting-based (CSUM-based) imaging approach that reorients the Z-axis into the high-resolution XY-plane using standard confocal microscopy, enabling direct RMT visualization without computational reconstruction or specialized hardware. We tracked intracellular trafficking of transferrin (Tf) and anti-transferrin receptor antibody (anti-TfR Ab) as model cargos using our CSUM-based imaging approach via compartment-specific markers and time-resolved co-localization analysis. This approach resolved cargo-containing vesicles traversing from the apical to basolateral membranes. Tf completed transcytosis within 15 min, whereas anti-TfR Ab initially entered the endolysosomal pathway before rerouting to transcytosis under receptor saturation conditions. The CSUM approach provides a simple yet effective platform for high-resolution visualization of membrane transport and vesicle dynamics, offering broad applicability to drug delivery research and the design of brain-targeted therapeutics.

## Graphical abstract

**Figure:**
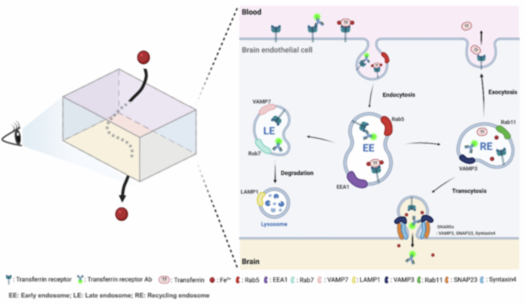

Schematic representation of the cell swelling and upright mounting (CSUM)–based imaging approach for high-resolution visualization of intracellular trafficking across the blood–brain barrier (BBB) using conventional confocal microscopy. Created with BioRender.com.

## 1. Introduction

The blood–brain barrier (BBB) is a highly specialized and selective interface between the peripheral vasculature and the central nervous system (CNS), maintaining neural homeostasis through stringent molecular regulation (Daneman and Prat 2015; Obermeier et al. 2013; Pardridge 1993; Wu et al. 2023; Zhao et al. 2015). It is formed by brain microvascular endothelial cells interconnected by tight junctions, which restrict the passage of ions, nutrients, and macromolecules (Abbott 2013; Daneman and Prat 2015; Obermeier et al. 2013; Sweeney et al. 2019; Terstappen et al. 2021). While essential for neuroprotection, the BBB severely limits CNS drug delivery, blocking ~98% of small molecules and virtually all biologics (Pandit et al. 2020; Sweeney et al. 2019; Wu et al. 2023). Under these constraints, receptor-mediated transcytosis (RMT) has emerged as a key mechanism for non-invasive delivery of macromolecular therapeutics, including proteins, peptides, and antibodies, across the BBB (Jones and Shusta 2007; Sweeney et al. 2019).

The ability to directly visualize RMT pathways at the subcellular level is crucial for understanding the fundamental mechanisms underlying BBB transport and for developing more effective brain-targeted therapeutics (Villaseñor et al. 2017). Real-time tracking of cargo transport across the polarized endothelial layer can provide critical insights into sequential trafficking events, compartment-specific interactions, and potential bottlenecks affecting transport efficiency (Ayloo and Gu 2019).

Given the importance of real-time, subcellular-level visualization of RMT pathways for elucidating BBB transport mechanisms and optimizing brain-targeted therapeutics, super-resolution techniques such as Stimulated Emission Depletion (STED) (Jahr et al. 2020) and Single-Molecule Localization Microscopy (SMLM) (Betzig et al. 2006; Khater et al. 2020; Lelek et al. 2021) are capable of achieving nanometer-scale resolution. However, they require costly and technically demanding setups, and their performance is often limited by the extremely thin (~200 nm) apicobasal thickness of brain endothelial cells (Lelek et al. 2021; Tian et al. 2020; Worzfeld and Schwaninger 2016).

This constraint reduces vertical resolution and hampers visualization of sequential trafficking events between the apical and basolateral membranes.

Conventional confocal microscopy combined with z-stack acquisition and three-dimensional (3D) reconstruction can partially address these issues, but axial (Z-axis) resolution remains substantially lower than lateral (XY-plane) resolution (Jonkman et al. 2020; Reilly and Obara 2021). While such limitations can be partially addressed using super-resolution microscopy or advanced 3D reconstruction techniques, these methods typically rely on expensive equipment and complex procedures, limiting their practical applicability (Schermelleh et al. 2010; Villaseñor and Collin 2017).

To overcome these limitations without relying on advanced imaging systems or complex computational workflows, we developed a cell swelling and upright mounting-based (CSUM-based) imaging approach. This method uses hypotonic cell swelling with 0.5X PBS (Jaiswal et al. 2019), and upright mounting of Transwell grown endothelial monolayers to convert low resolution Z-axis imaging into high resolution XY-plane visualization. This configuration enables direct observation of transcytotic vesicle movement using standard confocal microscopy, eliminating the need for computational reconstruction or advanced hardware.

To validate and demonstrate the capabilities of our novel imaging approach, we used transferrin (Tf) and anti-transferrin receptor antibodies (anti-TfR Ab) as model cargo molecules. Transferrin receptor 1 (TfR1), highly expressed on brain endothelial cells (Arguello and Mahon, 2022; Dufès et al. 2013; Li and Qian 2002), binds both native Tf and engineered anti TfR antibodies with high affinity, enabling saturable, receptor mediated transport across the BBB (Bien-Ly et al. 2014; Johnsen and Moos 2016; Wei et al. 2021). Their specificity and transport efficiency make them ideal molecular shuttles for evaluating RMT pathways (Moreira et al. 2024; Morrison et al. 2023; Petersen et al. 2024; Sela et al. 2023; Sonoda et al. 2022).

In this study, we introduce the CSUM-based approach for high-resolution visualization of intracellular trafficking across the BBB. Using Tf and anti-TfR Ab as model cargos, we tracked their trafficking dynamics in a physiologically relevant *in vitro* BBB monolayer via time-resolved co-localization with compartment-specific Rab GTPases (Ras-related guanosine triphosphate–binding proteins), including Rab5 for early endosome (EE), Rab7 for late endosome (LE), Rab11 for recycling endosome (RE), and SNARE proteins (soluble *N*-ethylmaleimide-sensitive factor attachment protein receptors), including vesicle-associated membrane protein 3 (VAMP3) for RE, vesicle-associated membrane protein 7 (VAMP7) for LE and syntaxin 4 and SNAP23 for basolateral membrane (BM) (Morad et al. 2019; Söllner et al. 1993). Thereby enabling direct assessment of RMT pathways. This strategy enabled high-resolution mapping of cargo movement along the apicobasal axis and provided mechanistic insights into the temporal and spatial organization of RMT, offering a broadly applicable platform for the study and optimization of brain-targeted drug delivery.

## 2. Materials and methods

### 2.1. Materials

Alexa Fluor® 647-conjugated mouse holo-transferrin (A647-Tf) was purchased from Jackson ImmunoResearch (PA, USA, cat. no. 015-600-050). Alexa Fluor® 647-conjugated anti-mouse transferrin receptor antibody (A647-anti-TfR Ab) was purchased from NOVUS Biologicals (CO, USA, cat. no. NB100-64979AF647). Transwell inserts with 0.4 μm pore size polyester membranes for 24-well and 6-well were purchased from Corning Falcon (MO, USA, cat. no. 353095 and 353090), Confocal *μ*-Dishes were purchased from ibidi GmbH (Gräfelfing, Germany, 35 mm, high glass bottom, cat. no. 81156), Poly-L-lysine hydrobromide was purchased from Sigma-Aldrich (MO, USA, cat. no. P4832). Dulbecco’s phosphate-buffered saline without calcium chloride (Ca²⁺) and magnesium chloride (Mg²⁺) (PBS) was purchased from WELGENE (Gyeongsan, Korea, cat. no. LB001-02). Other buffers and analytical-grade chemicals were obtained from J.T. Baker (PA, USA), TCI (Tokyo, Japan) and SIGMA Aldrich (MO, USA). All media-related reagents for cell culture were purchased from WELGENE (Gyeongsan, Korea). The mouse brain endothelial cell line bEnd.3 was obtained from ATCC. Hoechst 33342 (cat. no. H1399) and CellMask™ Orange (cat. no. C10045) were purchased from Invitrogen (NY, USA). All the primary and secondary antibodies were obtained from Abcam (NY, USA), Cell Signaling Technology (MA, USA) and ABclonal (MA, USA). List of rabbit anti-mouse primary and goat anti-rabbit secondary antibodies used in this study presents in Table 1.

**Table 1.** List of rabbit anti-mouse primary and goat anti-rabbit secondary antibodies used in this study.

Target protein	Subcellular compartment	Dilution	Supplier	Cat. no.
Early Endosome Antigen 1 (EEA1)	Early Endosome (EE)	1:1000	Cell Signaling Technology	3288S
Rab5	Early Endosome (EE)	1:1000	Cell Signaling Technology	3547S
Rab11	Recycling Endosome (RE)	1:1000	ABclonal	A3251
Vesicle-associated membrane protein 3 (VAMP3)	Recycling Endosome (RE)	1:1000	ABclonal	A8812
Rab7	Late Endosome (LE)	1:100	ABclonal	A12308
VAMP7	Late Endosome (LE)	1:1000	ABclonal	A18698
Lysosomal-associated membrane protein 1 (LAMP1)	Lysosome (Ly)	1:1000	Cell Signaling Technology	99437S
Synaptosomal-associated protein 23 (SNAP23)	Basolateral Membrane (BM)	1:1000	ABclonal	A13909
Syntaxin 4	Basolateral Membrane (BM)	1:1000	ABclonal	A5996
Alexa Fluor® 488 IgG H&L	Secondary antibody	1:1000	Abcam	Ab150077

### 2.2. Methods

#### 2.2.1. *In vitro* BBB permeability of Tf and anti-TfR Ab using BBB Transwell model

The transcytosis efficiency of Tf and anti-TfR Ab was evaluated using a 24-well Transwell BBB model. bEnd.3 cells were cultured on 0.4 μm pore polyester membrane inserts (0.33 cm²) in DMEM supplemented with 10% FBS and 1% penicillin streptomycin (P/S) at 37 °C, 5% CO₂, for > 5 days. Medium was replaced daily. The integrity of the BBB model was confirmed by measuring trans-endothelial electrical resistance (TEER) using EVOM2 (World Precision Instruments, Sarasota, FL, USA). TEER values above 250 Ω·cm² indicated a functional barrier. Before the assay, both apical and basolateral chambers were washed with PBS. The basolateral chamber was filled with 500 μL Ringer-HEPES buffer (RHB). A647-Tf and A647-anti-TfR Ab were diluted in RHB to 25 μg/mL and added to the apical chamber. Each condition was tested in triplicate (*n* = 3).

Basolateral samples (500 μL) were collected at 30 min, 1 h, 2 h, and 4 h, replacing each removed volume with fresh buffer. After 4 h, the remaining apical solution was also collected. Samples were stored at 4 °C in the dark until analysis.

Fluorescence intensity was measured with a SpectraMax® iD3 Multi-Mode Microplate Reader (Molecular Devices, CA, USA, Ex = 620 nm, Em = 665 nm). Concentrations were calculated from standard curves prepared with serial tracer dilutions (10, 50, 100, 250, 500, and 1000 ng/mL). Transport efficiency was expressed as cumulative cargo transported over time.

The apparent permeability coefficient (P_app_) at each time point was calculated as:

$$p_{\mathrm{app}}=\frac{C_{\mathrm{receiver}}\;\cdot \;V_{\mathrm{receiver}}}{A\;\cdot \;t\;\cdot \;C_{\mathrm{donor},\mathrm{initial}}}$$

Where ${\text{C}}_{\mathrm{receiver}}$ is the concentration of the tracer in the basolateral chamber, $V_{\text{receiver}}$ is basolateral volume (500 μL), A is the membrane surface area (0.33 cm²), t is incubation time (min), and $C_{\mathrm{donor},\mathrm{initial}}$ is the initial apical concentration (25 μg/mL). All units were standardized before calculation.

#### 2.2.2. Enzyme-linked immunosorbent assay (ELISA)

To evaluate *in vitro* BBB permeability of Tf and anti-TfR Ab, samples collected from the basolateral chambers of the Transwell BBB model were analysed using ELISA. Mouse transferrin concentration was quantified using a commercial ELISA kit (Abcam, NY, USA, ab157724). Standards and samples (100 μL each) were added to the pre-coated wells and incubated for 30 min at room temperature (RT). After three washes with 1X wash buffer, 100 μL of 1X horseradish peroxidase (HRP)-conjugated detection antibody was added to each well and incubated for another 30 min in the dark at RT. Following three additional washes, 100 μL of TMB substrate solution was added and incubated for 10 min. The reaction was stopped by adding 100 μL of stop solution, and absorbance was measured immediately at 450 nm using a microplate reader (VersaMax™, Molecular Devices, CA, USA).

For detection of mouse transferrin receptor antibody, a sandwich ELISA was performed following the methodology described previous study (Morrison et al. 2023). Briefly, high-binding 96-well plates were coated overnight at 4 °C with anti-mouse Fab-specific capture antibody (Jackson ImmunoResearch, PA, USA) in carbonate-bicarbonate buffer (pH 9.6). After blocking with 1% bovine serum albumin (BSA), samples were added and incubated, followed by treatment with HRP-conjugated goat anti-rat IgG secondary antibody. Absorbance at 450 nm was measured using a VersaMax™ Microplate Reader.

#### 2.2.3. Optimization of cell swelling conditions using a hypotonic solution

bEnd.3 cells (4 × 10⁵ cells/well) were seeded on 6-well Transwell inserts pre-coated with poly-L-lysine and cultured to confluence. Hypotonic treatment was performed using Ca²⁺/Mg²⁺-free PBS at different dilutions: distilled water (0X PBS), 0.2X PBS, and 0.5X PBS, prepared by dilution with distilled water (100% DW).

For time-course analysis, cell morphology was observed via bright-field microscopy at 1, 5, 8, and 10 min after treatment. For confocal microscopy, cells were treated with 1X PBS (control) or 0.5X PBS for 10 min at RT, fixed with 5% formalin for 10 min, and washed three times with PBS. Plasma membranes were stained with CellMask™ Orange (1 μg/mL) for 5 min, followed by three washes with PBS. Nuclei were subsequently stained with Hoechst 33342 (1 μg/mL) for 1 min. Confocal images were acquired using a laser scanning confocal microscope (LSCM, Nikon A1Plus, Tokyo, Japan).

Cell height was measured from apical to basolateral surfaces using NIS-Elements software (Nikon, Tokyo, Japan). Measurements were performed on 13 individual cells, and results were expressed as mean ± standard error of the mean (SEM).

#### 2.2.4. Immunocytochemistry

bEnd.3 cells (4 × 10⁵ cells/well) were seeded on 6-well Transwell inserts pre-coated with poly-L-lysine and cultured to confluence. A647-Tf and A647-anti-TfR Ab were diluted in serum-free medium to 5 μg/mL and applied to cells for 5 min, 15 min, or 1 h.

After incubation, cells were washed three times with PBS and subjected to hypotonic swelling with 0.5X PBS for 10 min at RT. Cells were fixed with formalin for 10 min, permeabilized with 0.1% Triton X-100 for 5 min, and washed three times with PBS.

Primary antibodies against compartment-specific markers were applied for 1 h at RT: early endosome antigen 1 (EEA1) and Rab5 for early endosome (EE), Rab11 and VAMP3 for recycling endosome (RE), Rab7 and VAMP7 for late endosome (LE), lysosomal-associated membrane protein 1 (LAMP1) for lysosome (Ly), SNAP23 and syntaxin 4 for the basolateral membrane (BM) (Hong 2005; Morad et al. 2019; Toth et al. 2020).

After PBS washes, cells were incubated for 1 h with Alexa Fluor® 488-conjugated goat anti-mouse IgG H&L. Nuclei were counterstained with Hoechst 33342 (1 μg/mL, 1 min).

#### 2.2.5. Upright mounting process and laser scanning confocal microscopy (LSCM) imaging analysis

Following immunostaining, the Transwell membranes were carefully collected using a sterile scalpel and sliced into ~1 mm-wide strips. Approximately 1 mm at both ends was folded using forceps to allow upright positioning, and the strips were then mounted vertically in a confocal imaging dish containing 1 mL glycerol.

High-resolution apicobasal imaging was performed using LSCM (Nikon, A1Plus, Tokyo, Japan) equipped with a 60 × oil immersion objective lens (Apo 60 × oil λS DIC N2, numerical aperture = 1.40). Images were acquired at 512 × 512 pixels under a 12-bit dynamic range. The lateral and axial spatial resolutions were approximately 0.2–0.3 μm and 0.6–0.8 μm (Inoué, 2006). Both single-plane and z-stack images were acquired, and co-localization was quantified using Manders’ overlap coefficient in NIS-Elements software.

#### 2.2.6. Statistical analysis

Statistical analyses were performed using GraphPad Prism 8.0.1 (GraphPad Software, USA). Data are presented as mean ± SEM. Two-way ANOVA was used to evaluate interaction effects between carriers and time points. Statistical significance was set at *p* < 0.05 (*), *p* < 0.01 (**), *p* < 0.001 (***), and *p* < 0.0001 (****). Comparisons showing *p* ≥ 0.05 were indicated not significant (ns).

## 3. Results

### 3.1. *In vitro* BBB permeability of Tf and anti-TfR Ab

Before assessing intracellular trafficking, we first validated the transport capacity of the *in vitro* BBB model. Time-course transport assays showed progressive accumulation of both Tf and anti-TfR Ab in the basolateral chamber over 4 h (Table S1–S4). Quantification was performed using two complementary readouts on the same Alexa Fluor® 647-labeled samples: (i) fluorescence measurement and (ii) ELISA detection with molecule-specific antibodies. Both methods yielded closely matching apparent permeability coefficient (P_app_) values at each time point. At 1 h, fluorescence- and ELISA-based measurements were 0.749 ± 0.002 and 0.757 ± 0.003 for anti-TfR Ab, and 2.167 ± 0.003 and 2.154 ± 0.002 for Tf (cm/s × 10^−6^), respectively, confirming high concordance between methods. The obtained P_app_ values were consistent with receptor-mediated transport characteristics previously reported for TfR-mediated BBB transcytosis, validating the suitability of this model for subsequent trafficking analyses (Broadwell et al. 1996; Sade et al. 2014; Thomsen et al. 2021).

TEER values remained ≥ 250 Ω·cm² both before and after the experiments (Figure S1), indicating that the tight junction integrity of the BBB monolayer was preserved throughout the assays. This validated the model for subsequent trafficking analyses.

### 3.2. Cell swelling by hypotonic solution

**Figure 1.**
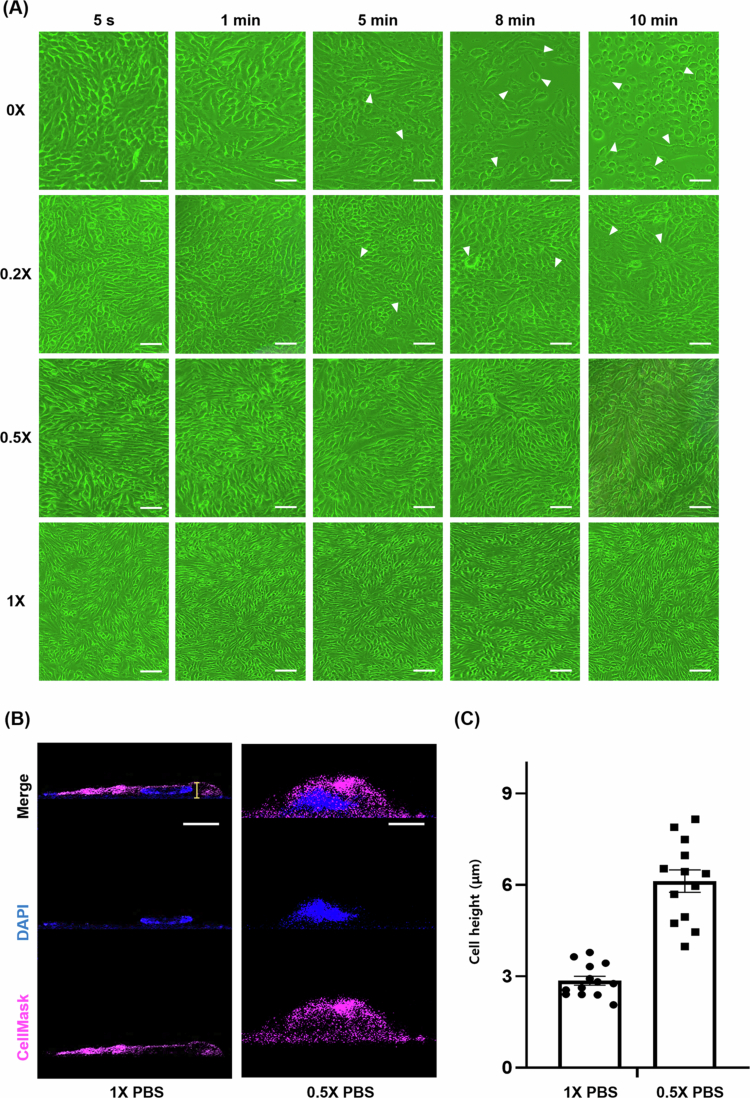
Hypotonic treatment optimization for enhanced intracellular visualization. (A) Optical micrographs of cells treated with three hypotonic solutions prepared by mixing distilled water (DW) and PBS—pure DW (0X, 100% DW), 0.2X PBS solution, and 0.5X PBS solution. Cells were imaged at 1, 5, 8 and 10 min post-treatment. White arrows indicate representative cells showing membrane rupture or cellular stress under stronger hypotonic conditions (0X and 0.2X PBS). Scale bar: 50 μm. (B) Confocal microscopy images of bEnd.3 cells after hypotonic treatment showing cell morphology with DAPI (blue) and cell mask staining. Left panel: 1X PBS control treatment for 10 min. Right panel: 0.5X PBS treatment for 10 min. Scale bar: 10 μm. (C) Quantitative analysis of cell height measured using NIS-E software program following 10 min treatment with 1X PBS (control) versus 0.5X PBS hypotonic solution. Data are presented as mean ± SEM (*n* = 13).

We evaluated the effects of hypotonic treatment on bEnd.3 cell morphology to enhance imaging resolution (Figure 1A). Cells treated with 0.2X PBS or 0X PBS exhibited rapid and pronounced morphological alterations within 5 min, losing their elongated endothelial shape and becoming rounded. Both conditions led to extensive cell swelling accompanied by loss of membrane definition and signs of membrane rupture or cellular stress, particularly in the 0X group due to excessive osmotic pressure (white arrows in Figure 1A). In contrast, 0.5X PBS induced gradual and uniform cytoplasmic expansion over 10 min without compromising membrane integrity, maintaining overall cell shape suitable for high-resolution imaging.

Confocal microscopy (Figure 1B) revealed that 0.5X PBS increased cytoplasmic volume and enhanced organelle separation, as confirmed by CellMask™ Orange membrane staining and Hoechst nuclear staining. Quantitative analysis showed that the average cell height increased from 2.853 ± 0.148 μm under isotonic (1X PBS) conditions to 6.123 ± 0.367 μm after 0.5X PBS treatment, representing approximately a two-fold increase (*n* = 13; Mean ± SEM; Figure 1C). Importantly, TEER measurements confirmed that this hypotonic treatment did not compromise barrier integrity, with stable resistance maintained throughout the 10 min exposure (Figure S2). Based on these results, 0.5X PBS for 10 min was selected as the optimal swelling condition for CSUM imaging.

### 3.3. CSUM-based imaging enables direct visualization of transcytotic transport

**Figure 2.**
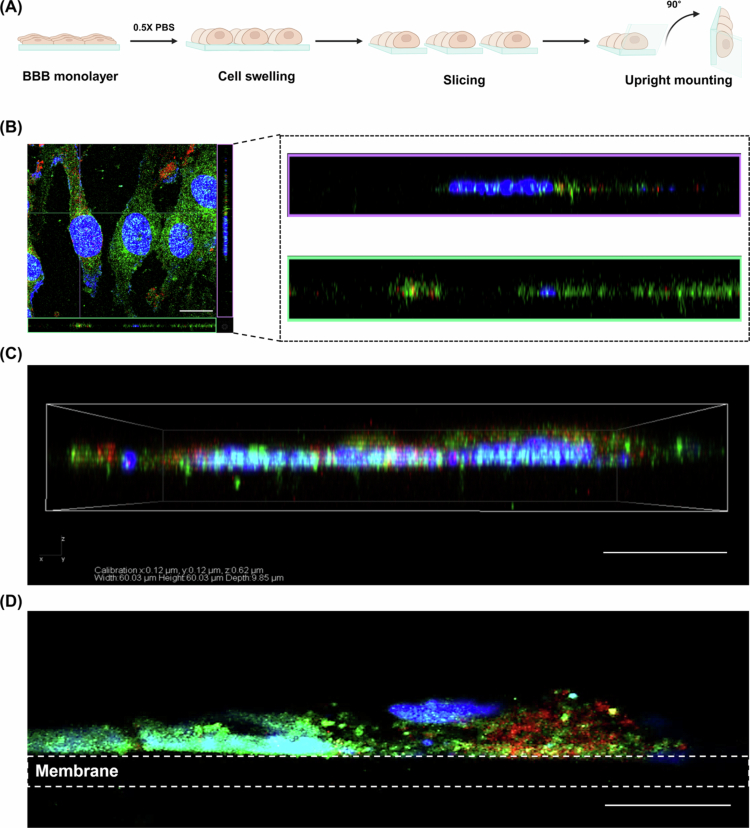
Comparative visualization of conventional z-stack imaging and the CSUM-based imaging approach for transcytosis analysis. (A) Schematic illustration of CSUM-based imaging approach. Created with BioRender.com. (B)–(C) 3D reconstruction of z-stacked confocal images acquired in the conventional XY-plane (red: anti-TfR Ab, green: SNAP23, blue: DAPI). Scale bar: 25 μm. (D) Confocal image acquired from the apicobasal plane using the configuration shown in (A) (red: anti-TfR Ab, green: SNAP23, blue: DAPI). Scale bar: 10 μm.

Our novel CSUM-based approach, which integrates hypotonic cell swelling, precise membrane slicing, and upright mounting, markedly enhanced spatial resolution along the apicobasal axis. As illustrated in Figure 2A, this sequence of preparatory steps reoriented the endothelial monolayer for vertical imaging using standard confocal microscopy, eliminating the need for computational reconstruction or specialized optics.

Figure 2B–C illustrate the limitations of conventional z-stack imaging, where low axial resolution blurs compartment boundaries and obscures vesicular details. Based on the pixel dimensions measured for a representative region of interest (ROI; 10 μm × 5 μm), the ROI in Figure 2B–C spans 24 × 14 pixels, whereas the same-sized ROI in Figure 2D spans 83 × 42 pixels. Thus, CSUM-based imaging analysis enables high-resolution visualization of cells in the apicobasal view, thereby enhancing the apparent delineation of vesicle contours, organelle boundaries, and membrane interfaces along the axis most relevant to transcytotic progression. These advantages reflect improved digital sampling for visualization rather than any change in the intrinsic optical resolution of the confocal system.

### 3.4. Distinct temporal trafficking patterns of Tf and anti-TfR Ab through endosomal compartments

**Figure 3.**
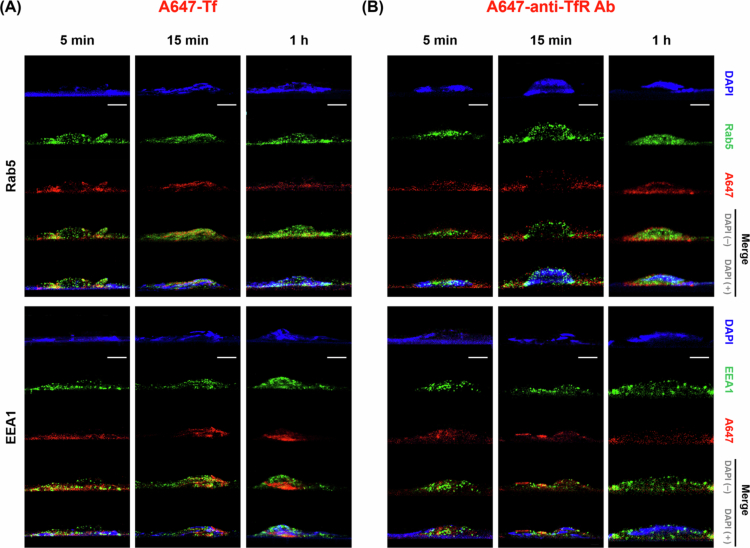
Confocal microscopy analysis of early endosome trafficking patterns. (A) Cells treated with A647-Tf for 5 min, 15 min, or 1 h, stained with DAPI (blue), Rab5 (green, upper panels), and EEA1 (green, lower panels). (B) Cells treated with A647-anti-TfR Ab under the same conditions and staining. The red signal corresponds to internalized A647-Tf or A647-anti-TfR Ab. Scale bar: 10 μm.

**Figure 4.**
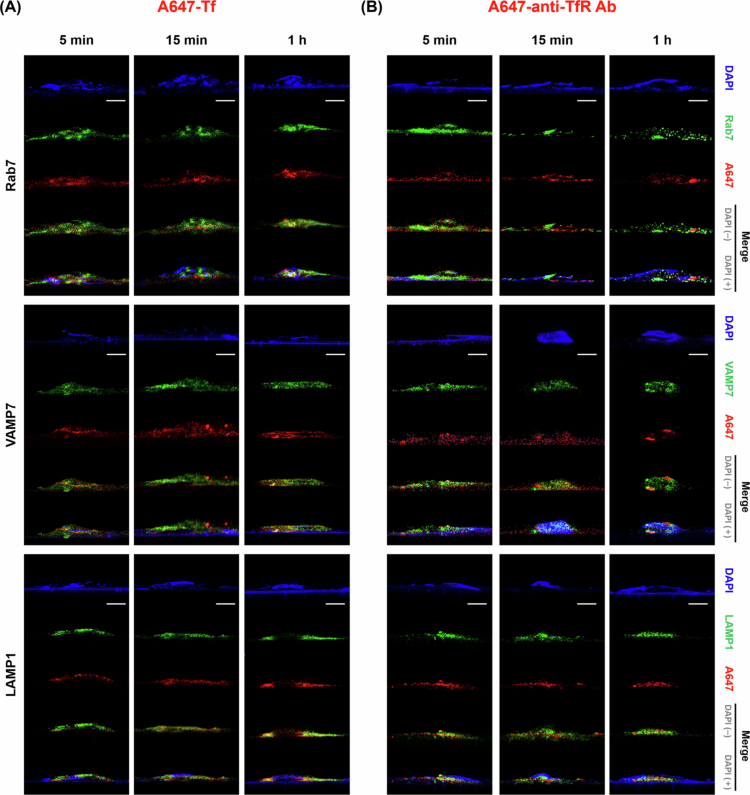
Late endosome and lysosomal trafficking analysis. (A) Cells treated with A647-Tf for 5 min, 15 min, or 1 h, stained with DAPI (blue), Rab7 (green, upper panels), VAMP7 (green, middle panels), and LAMP1 (green, lower panels). (B) Cells treated with A647-anti-TfR Ab under the same conditions and staining. The red signal corresponds to internalized A647-Tf or A647-anti-TfR Ab. Scale bar: 10 μm.

**Figure 5.**
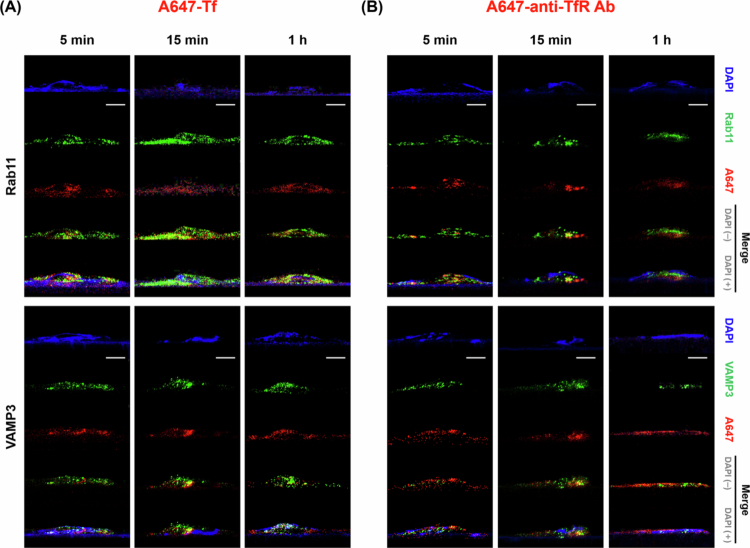
Recycling endosome trafficking analysis. (A) Cells treated with A647-Tf for 5 min, 15 min, or 1 h, stained with DAPI (blue), Rab11 (green, upper panels), and VAMP3 (green, lower panels). (B) Cells treated with A647-anti-TfR Ab under the same conditions and staining. The red signal corresponds to internalized A647-Tf or A647-anti-TfR Ab. Scale bar: 10 μm.

**Figure 6.**
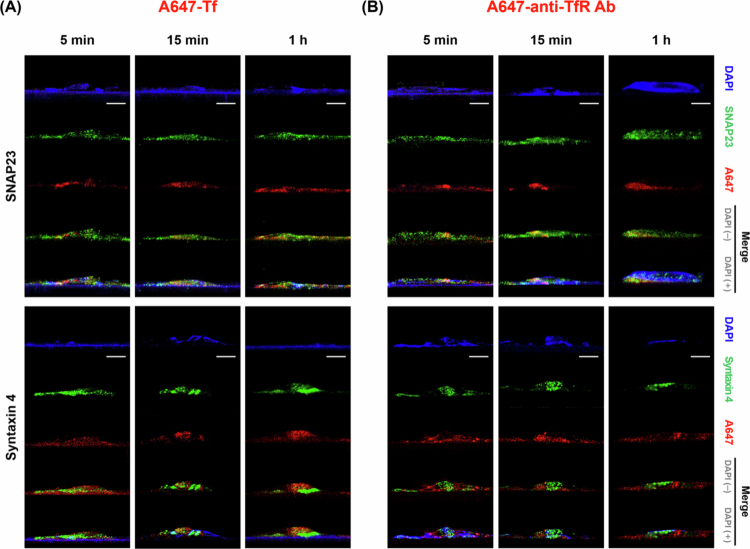
Basolateral membrane fusion analysis. (A) Cells treated with A647-Tf for 5 min, 15 min, or 1 h, stained with DAPI (blue), SNAP23 (green, upper panels), and syntaxin 4 (green, lower panels). (B) Cells treated with A647-anti-TfR Ab under the same conditions and staining. The red signal corresponds to internalized A647-Tf or A647-anti-TfR Ab. Scale bar: 10 μm.

**Figure 7.**
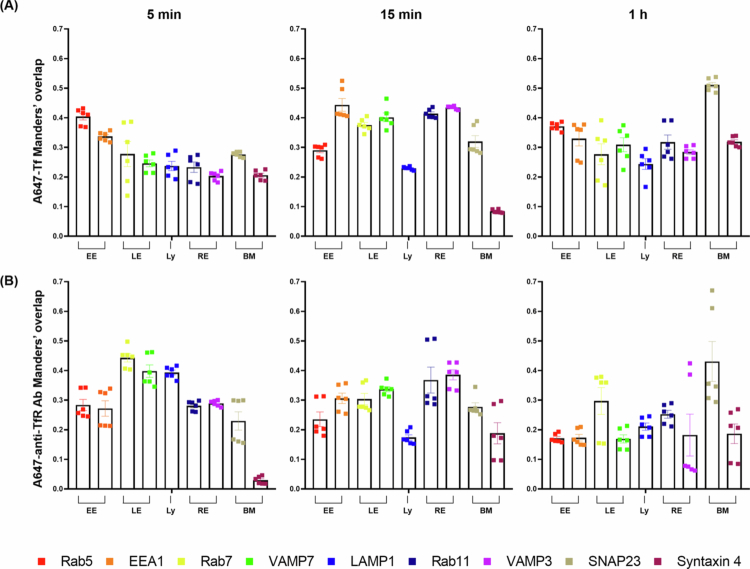
Co-localization analysis of A647-Tf (A) and A647-anti-TfR Ab (B) with intracellular markers over time. Manders’ overlap coefficients were calculated from multiple xy sections within z-stack confocal images acquired using NIS-Elements (NIS-E) software. Cells were treated with A647-Tf or A647-anti-TfR Ab (5 μg/mL) for 5 min, 15 min, or 1 h, and stained with markers for early endosomes (EE: Rab5, EEA1), late endosomes (LE: Rab7, VAMP7), lysosomes (Ly: LAMP1), recycling endosomes (RE: Rab11, VAMP3), and basolateral membrane (BM: SNAP23, syntaxin 4). Data are presented as mean ± SEM (*n* = 6, each image was obtained from one sample in a single experiment).

By monitoring Tf and anti-TfR Ab trafficking at multiple time points (5 min, 15 min, and 1 h), we mapped their progression through EE, LE-Ly, RE and finally the BM. Co-localization was identified when the red fluorescence signal (model cargo) overlapped with the green signal (EE, LE-Ly, RE, and BM markers), appearing as yellow or orange in the merged images Figures 3–6. Representative conventional confocal XY-plane images of Tf and anti-TfR Ab with compartment-specific markers are included in the Supplementary Information (Figure S3–S10). The quantitative analysis of these co-localization events, derived from our custom vertical confocal imaging approach that enabled direct visualization of transcellular transport along the apicobasal axis of endothelial cells, is presented as bar graphs in Figure 7, with corresponding numerical values summarized in Tables 2, 3. The overall carrier × time interaction effects obtained from two-way ANOVA are summarized in Table S5.

**Table 2.** Manders’ overlap coefficients for A647-Tf co-localization with compartment-specific markers at 5 min, 15 min, and 1 h. (*n* = 6, mean ± SEM).

Marker	5 min	15 min	1 h
Rab5	0.404 ± 0.011	0.290 ± 0.008	0.371 ± 0.006
EEA1	0.337 ± 0.006	0.444 ± 0.022	0.330 ± 0.025
Rab7	0.278 ± 0.042	0.376 ± 0.009	0.277 ± 0.035
VAMP7	0.246 ± 0.011	0.402 ± 0.015	0.309 ± 0.023
LAMP1	0.237 ± 0.016	0.229 ± 0.002	0.244 ± 0.018
Rab11	0.233 ± 0.017	0.414 ± 0.006	0.318 ± 0.024
VAMP3	0.203 ± 0.006	0.434 ± 0.002	0.284 ± 0.008
SNAP23	0.276 ± 0.004	0.320 ± 0.020	0.511 ± 0.009
Syntaxin 4	0.206 ± 0.007	0.084 ± 0.002	0.319 ± 0.007

**Table 3.** Manders’ overlap coefficients for A647-anti-TfR Ab co-localization with compartment-specific markers at 5 min, 15 min, and 1 h. (*n* = 6, mean ± SEM).

Marker	5 min	15 min	1 h
Rab5	0.284 ± 0.019	0.235 ± 0.025	0.171 ± 0.006
EEA1	0.272 ± 0.026	0.306 ± 0.018	0.173 ± 0.011
Rab7	0.443 ± 0.014	0.304 ± 0.019	0.297 ± 0.045
VAMP7	0.398 ± 0.021	0.337 ± 0.009	0.169 ± 0.014
LAMP1	0.394 ± 0.008	0.174 ± 0.009	0.211 ± 0.012
Rab11	0.281 ± 0.007	0.368 ± 0.044	0.252 ± 0.013
VAMP3	0.289 ± 0.004	0.386 ± 0.017	0.182 ± 0.071
SNAP23	0.230 ± 0.031	0.277 ± 0.014	0.431 ± 0.068
Syntaxin 4	0.030 ± 0.005	0.188 ± 0.036	0.186 ± 0.033

#### 3.4.1. Early endosome association patterns differ between Tf and anti-TfR Ab

Co-localization with EE markers Rab5 and early endosome antigen 1 (EEA1) revealed distinct initial trafficking patterns for Tf and anti-TfR Ab. At 5 min, Tf showed high co-localization with Rab5 (0.404 ± 0.011) and EEA1 (0.337 ± 0.006), indicating efficient internalization into EE. In contrast, anti-TfR Ab displayed lower co-localization with Rab5 (0.284 ± 0.019) and EEA1 (0.272 ± 0.026) at the same time point, suggesting that a significant fraction of antibody-containing vesicles progressed beyond the EE stage soon after uptake. By 15 min, Tf showed reduced Rab5 (0.290 ± 0.008), while EEA1 co-localization further increased (0.444 ± 0.022), whereas anti-TfR Ab showed Rab5 co-localization comparable to the 5 min level (0.235 ± 0.025) and slightly increased association with EEA1 (0.306 ± 0.018). At 1 h, Tf maintained relatively high co-localization with Rab5 (0.371 ± 0.006) and EEA1 (0.330 ± 0.025). This pattern may suggest that a substantial fraction of Tf continues to interact with the EE compartment at this time point. In contrast, anti-TfR Ab showed a progressive reduction in both Rab5 (0.171 ± 0.006) and EEA1 (0.173 ± 0.011) signals, indicating sustained departure from the EE compartment. In merged images, these trends were visually represented by stronger yellow/orange signal intensity for Tf compared with anti-TfR Ab at early time points (Figure 3).

#### 3.4.2. Late endosome/Lysosome trafficking profiles diverge between Tf and anti-TfR Ab

At 5 min, anti-TfR Ab exhibited markedly higher co-localization with LE markers Rab7 (0.443 ± 0.014) and VAMP7 (0.398 ± 0.021), compared to Tf (Rab7: 0.278 ± 0.042; VAMP7: 0.246 ± 0.011). By 15 min, anti-TfR Ab co-localization with LE markers decreased (Rab7: 0.304 ± 0.019; VAMP7: 0.337 ± 0.009), whereas Tf increased LE association (Rab7: 0.376 ± 0.009; VAMP7: 0.402 ± 0.015). At 1 h, LE marker association was lower overall than at 15 min for both cargos (Tf: Rab7 0.277 ± 0.035, VAMP7 0.309 ± 0.023; anti-TfR Ab: Rab7 0.297 ± 0.045, VAMP7 0.169 ± 0.014). For the Ly marker LAMP1, anti-TfR Ab showed higher association than Tf at 5 min (0.394 ± 0.008 vs. 0.237 ± 0.016), indicating early routing to late endosomal compartments. At 15 min, LAMP1 association was comparable between the two cargos (Tf: 0.229 ± 0.004; anti-TfR Ab: 0.174 ± 0.009), and for Tf it was also comparable to the 5 min level (0.237 ± 0.016). At 1 h, LAMP1 association was comparable to or slightly higher than at 15 min (Tf: 0.244 ± 0.018 vs. 0.229 ± 0.004; anti-TfR Ab: 0.211 ± 0.012 vs. 0.174 ± 0.009) (Figure 4).

#### 3.4.3. Recycling endosome routing patterns differ between Tf and anti-TfR Ab

Recycling endosome analysis using Rab11 and VAMP3 revealed divergent time-dependent patterns. Tf maintained moderate RE association at 5 min (Rab11: 0.233 ± 0.017, VAMP3: 0.203 ± 0.006), increasing over time to Rab11: 0.414 ± 0.006 and VAMP3: 0.434 ± 0.002 at 15 min, then slightly decreasing at 1 h (Rab11: 0.318 ± 0.024, VAMP3: 0.284 ± 0.008). anti-TfR Ab initially showed lower Rab11 (0.281 ± 0.007) and VAMP3 (0.289 ± 0.004) co-localization, but displayed a marked increase by 15 min (Rab11: 0.368 ± 0.044, VAMP3: 0.386 ± 0.017), concurrent with reduced LE-Ly association, before declining again at 1 h (Rab11: 0.252 ± 0.013, VAMP3: 0.182 ± 0.017). Overall, both ligands showed the strongest yellow/orange signal at 15 min, which decreased at 1 h, with higher RE association for Tf at 15 min (Figure 5).

#### 3.4.4. Basolateral fusion dynamics reveal differences in transcytosis completion between Tf and anti-TfR Ab

Co-localization with the t-SNARE SNAP23 and syntaxin 4 at the BM highlighted differences in transcytosis completion. Tf showed higher SNAP23 association than anti-TfR Ab across all time points (5 min: 0.276 ± 0.004 vs. 0.230 ± 0.031; 15 min: 0.320 ± 0.020 vs. 0.277 ± 0.014; 1 h: 0.511 ± 0.009 vs. 0.431 ± 0.068). Tf showed the highest co-localization with syntaxin 4 at 1 h (5 min: 0.206 ± 0.007; 15 min: 0.084 ± 0.002; 1 h: 0.319 ± 0.007). anti-TfR Ab showed low syntaxin 4 co-localization across all time points (5 min: 0.030 ± 0.005; 15 min: 0.188 ± 0.036; 1 h: 0.186 ± 0.033). Overall, these patterns may suggest that Tf is more likely than anti-TfR Ab to engage basolateral fusion events associated with transcytosis completion. This trend was qualitatively illustrated in the merged images, while comparisons across ligands and time points were supported by the quantitative co-localization analysis (Figure 6).

## 4. Discussion

The CSUM-based imaging approach provided a simple yet powerful solution to overcome the inherent axial resolution limitations of conventional confocal microscopy when studying receptor-mediated transcytosis across the BBB. By combining hypotonic cell swelling, precise membrane slicing, and upright mounting of Transwell-grown endothelial monolayers, this method reorients the apicobasal axis into the high resolution XY-plane, enabling direct visualization of vesicular trafficking without the need for super-resolution hardware or computational reconstruction. This streamlined configuration offers several advantages: (i) it preserves the native architecture of polarized endothelial monolayers, (ii) it allows rapid implementation using widely available equipment, and (iii) it yields high spatial and temporal resolution sufficient to map cargo progression through distinct endosomal compartments and basolateral fusion events. These strengths make the CSUM approach broadly applicable for mechanistic dissection of BBB transport and for comparative screening of brain-targeted therapeutics under physiologically relevant conditions.

Using this approach, intracellular trafficking of Tf and anti-TfR Ab was tracked under identical conditions. Time-resolved co-localization with compartment-specific Rab GTPases and SNARE proteins mapped sequential progression from early endosomes to late endosome–lysosome or recycling compartments, followed by basolateral membrane fusion. High spatial and temporal resolution enabled direct comparison of trafficking dynamics and identification of distinct routing patterns between the two cargos.

Tf exhibited a streamlined trafficking pathway characterized by rapid entry into early endosomes (EE), minimal engagement with late endosome–lysosome (LE–Ly) compartments, progressive accumulation in recycling endosomes (RE), and timely SNARE complex formation at the basolateral membrane (BM). This sequence was completed within 15 min, indicating efficient transcytosis across the BBB endothelial layer with minimal intracellular retention, similar to the well-established transport pathway of transferrin.

In contrast, anti-TfR Ab displayed an atypical early trafficking pattern, showing predominant association with LE–Ly markers immediately after uptake, exceeding EE co-localization at 5 min. This initial routing may reflect differences in receptor binding kinetics, avidity, or intracellular sorting signals intrinsic to the antibody structure. Subsequent trafficking involved a delayed shift toward recycling compartments, accompanied by gradual but incomplete SNARE assembly at the BM. Even at 1 h, syntax in 4 co-localization remained substantially lower than that of Tf, suggesting prolonged retention in intermediate compartments and slower completion of transcytosis. The observed retention of anti-TfR Ab within late endosome–lysosome compartments may, at least in part, reflect differences in receptor–ligand binding affinity. It has been reported that the intracellular fate of TfR-targeted antibodies depends strongly on their interaction strength with TfR: moderate-affinity antibodies tend to dissociate and recycle or undergo transcytosis, whereas high-affinity antibodies remain bound and are directed toward lysosomal degradation (Arguello et al. 2022; Bien-Ly et al. 2014). Therefore, the slower and less complete transcytosis observed for anti-TfR Ab in this study may be attributable to such affinity-dependent sorting behavior. Future studies comparing antibodies with defined affinity variants could clarify how receptor binding strength influences trafficking fate across the BBB.

Importantly, the CSUM-based approach proved effective in detecting these pathway differences at high spatial and temporal resolution without specialized hardware. By providing compartment-specific trafficking maps over defined time intervals, this method can serve as a powerful platform for mechanistic studies and for screening BBB-targeted therapeutics. The ability to directly compare trafficking routes of different cargos under identical conditions enhances its value for rational design of brain drug delivery strategies.

Several limitations should be acknowledged in this study. While a set of widely used and well-characterized compartment-specific markers, including Rab GTPases and SNARE proteins, was employed, the molecular machinery governing intracellular trafficking is considerably more complex than these markers can capture. The endosomal system involves numerous additional regulatory proteins, including other Rab family members, sorting nexins, endosomal sorting complexes required for transport (ESCRT) complexes, and various adaptor proteins that fine-tune vesicle trafficking decisions (Cullen and Korswagen 2012; Stenmark 2009). Moreover, the dynamic interplay between different SNARE isoforms, their regulatory proteins (such as Munc18, complexins, and synaptotagmins), and the temporal coordination of membrane fusion events involves intricate molecular cascades that extend beyond the resolution of co-localization analysis (Baker and Hughson 2016; Südhof 2013). Our approach, while informative for mapping general trafficking routes, cannot resolve the detailed molecular interactions, protein complex formation kinetics, or precise temporal sequences of regulatory events that govern vesicle fate (Kusumi et al. 2012; Söderberg et al. 2006).

In addition, the *in vitro* BBB model cannot fully reproduce the physiological complexity of the *in vivo* neurovascular unit, where brain endothelial trafficking is shaped by hemodynamic forces, perivascular cell interactions, and glial crosstalk. These modulators, together with factors such as blood flow, shear stress, and neuroinflammatory states, can alter vesicular routing, potentially leading to differences from *in vitro* observations. Furthermore, the composition of the assay medium used in this study represents a simplified environment that mimics the ionic balance of plasma but lacks its complex protein and lipid components. This controlled setup enables precise analysis of RMT yet does not fully reflect the biochemical environment of brain endothelial cells *in vivo*. Such compositional differences may affect the absolute rate of transcytosis, although relative comparison under identical conditions remain valid. Applying the CSUM approach *in vivo* also poses technical and biological challenges, as the required steps of hypotonic swelling, membrane slicing, and upright mounting are incompatible with intact vascular networks. Although intravital imaging techniques such as two-photon microscopy may offer partial adaptation, achieving the same spatial resolution and apicobasal alignment obtained *in vitro* remains difficult in deep tissue.

Finally, the hypotonic swelling step, while essential for reorienting the imaging plane in our setup, carries the potential caveat of altering the native spatial organization of subcellular organelles. As shown by our TEER measurements (Figure S2), BBB integrity was preserved during the 10 min hypotonic exposure. The higher TEER in 0.5X versus 1X PBS, also observed in blank inserts, reflects reduced ionic strength–dependent conductivity rather than tighter BBB sealing or increased cell thickness. Nonetheless, Osmotic perturbations may induce subtle morphological shifts or positional changes that could influence co-localization readouts. Although the observed trafficking patterns are consistent and reproducible under our conditions, these potential effects should be considered when interpreting the data.

Future studies could extend the CSUM approach beyond the BBB monolayer to other barrier interfaces, including different endothelial types, multi-layered systems, and co-culture models with supporting cells. For animal models, the method could be adapted via intravital two-photon imaging of superficial microvessels or *ex vivo* reorientation of isolated microvessels to approximate the apicobasal axis, enabling *in vivo* mapping of transcytosis pathways and validation of *in vitro* findings. These extensions would facilitate investigation of how factors such as iron status, inflammation, and disease states modulate trafficking mechanisms, ultimately advancing drug delivery research.

## 5. Conclusions

In this study, we developed a cell swelling and upright mounting (CSUM)–based imaging approach that enables direct, high-resolution visualization of RMT across the apicobasal axis of an *in vitro* BBB model without specialized equipment or computational reconstruction. Using this method, we demonstrated that Tf completes rapid, recycling-oriented transcytosis within 15 min, whereas anti-TfR Ab is predominantly routed to degradative compartments before partial recycling. These results underscore that ligand properties fundamentally dictate trafficking fate and highlight CSUM as a practical and reproducible platform for elucidating transcytotic mechanisms and advancing brain drug delivery.

## Supplementary Material

second revision_Original image for microscopy studies.zip second revision_Original image for microscopy studies.zip

second_revision_20251216_supplementary_information Clean.docx second_revision_20251216_supplementary_information Clean.docx

## Data Availability

All data needed to evaluate the conclusions in the paper are present in the paper and/or the Supplementary Materials. The data that support the findings of this study are available from the corresponding author upon reasonable request.
