# Supplementary figures and images for: Cell swelling and upright mounting-based imaging for high-resolution visualization of intracellular trafficking across the BBB using conventional confocal microscopy (part 2 of 2)
Source: Drug Deliv. 2026 Jan 6;33(1):2608235. doi: 10.1080/10717544.2025.2608235 (PMC12781939; doi:10.1080/10717544.2025.2608235)

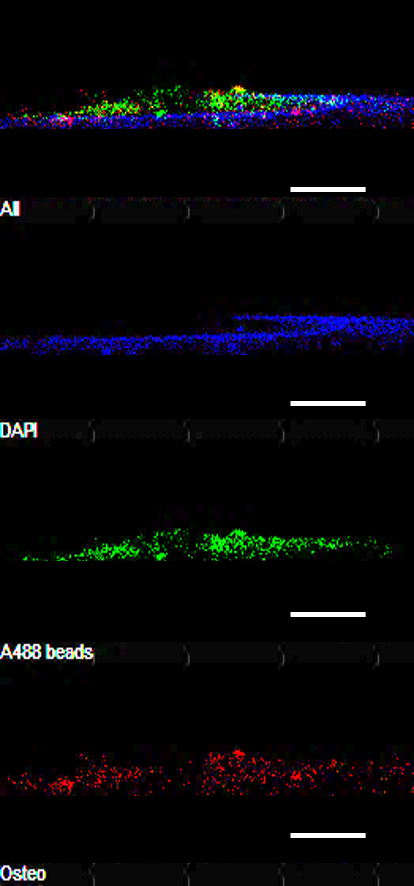

Supplement: second revision_Original image for microscopy studies.zip [file IDRD_A_2608235_SM4435.zip › Original Image for fig 004B_middle left panel.tif]

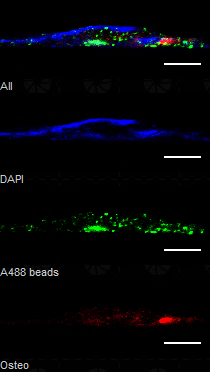

Supplement: second revision_Original image for microscopy studies.zip [file IDRD_A_2608235_SM4435.zip › Original Image for fig 004B_upper right panel.tif]

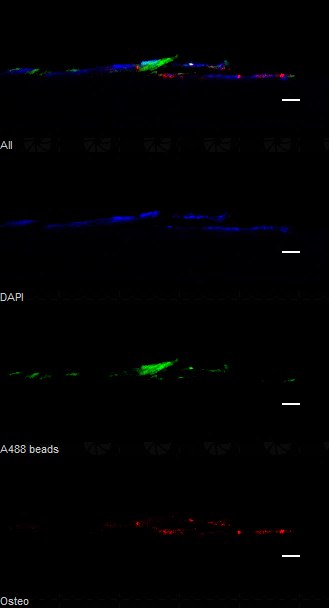

Supplement: second revision_Original image for microscopy studies.zip [file IDRD_A_2608235_SM4435.zip › Original Image for fig 004B_upper middle panel.tif]

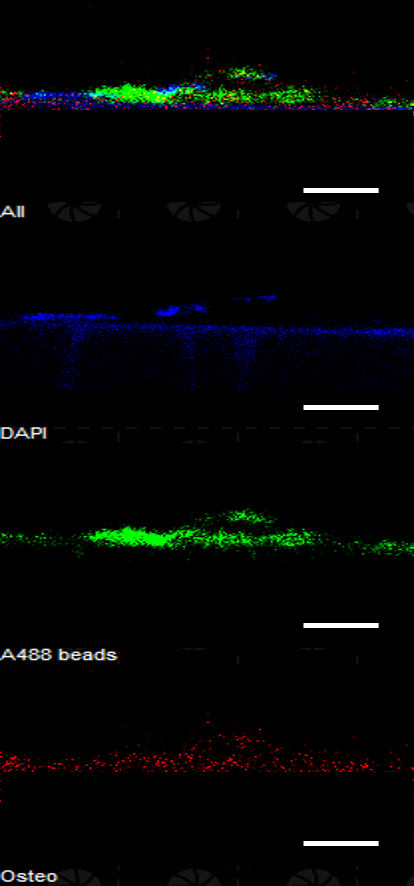

Supplement: second revision_Original image for microscopy studies.zip [file IDRD_A_2608235_SM4435.zip › Original Image for fig 004B_upper left panel.tif]

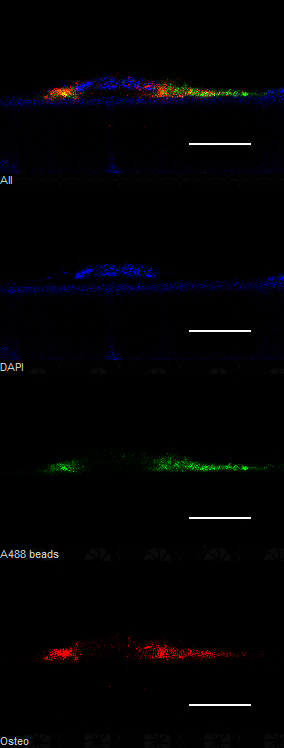

Supplement: second revision_Original image for microscopy studies.zip [file IDRD_A_2608235_SM4435.zip › Original Image for fig 004A_lower right panel.tif]

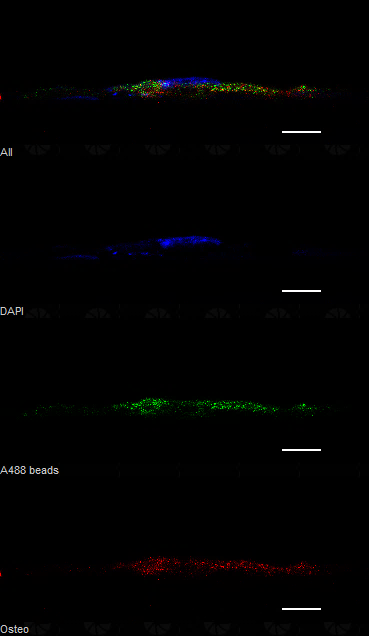

Supplement: second revision_Original image for microscopy studies.zip [file IDRD_A_2608235_SM4435.zip › Original Image for fig 004A_lower midle panel.tif]

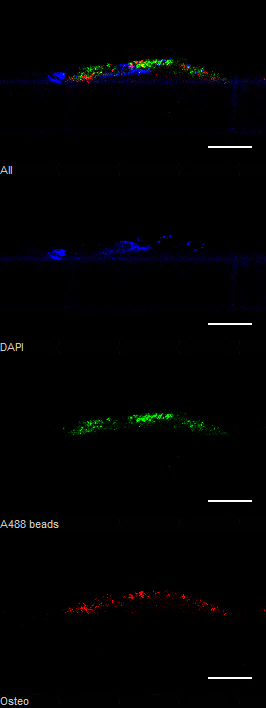

Supplement: second revision_Original image for microscopy studies.zip [file IDRD_A_2608235_SM4435.zip › Original Image for fig 004A_lower left panel.tif]

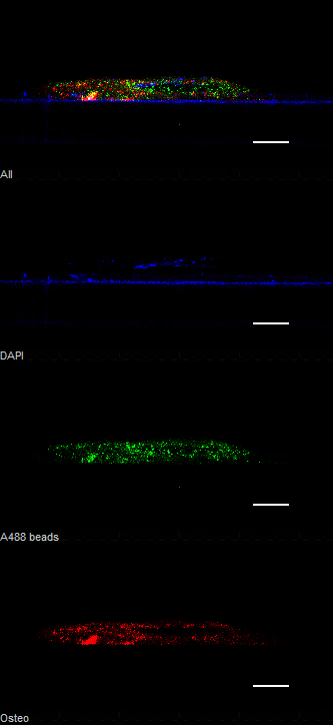

Supplement: second revision_Original image for microscopy studies.zip [file IDRD_A_2608235_SM4435.zip › Original Image for fig 004A_middle right panel.tif]

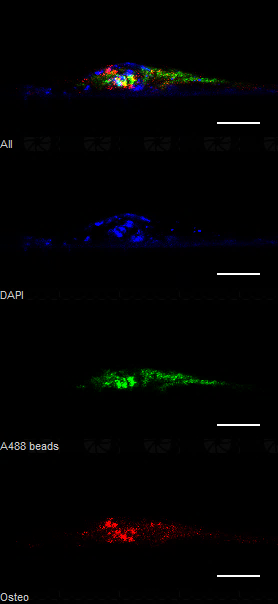

Supplement: second revision_Original image for microscopy studies.zip [file IDRD_A_2608235_SM4435.zip › Original Image for fig 004A_upper right panel.tif]

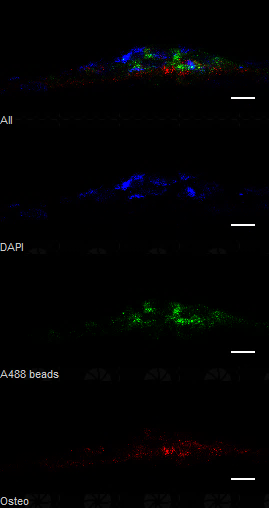

Supplement: second revision_Original image for microscopy studies.zip [file IDRD_A_2608235_SM4435.zip › Original Image for fig 004A_upper middle panel.tif]

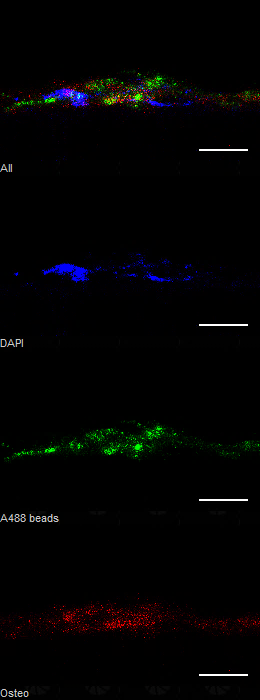

Supplement: second revision_Original image for microscopy studies.zip [file IDRD_A_2608235_SM4435.zip › Original Image for fig 004A_upper left panel.tif]

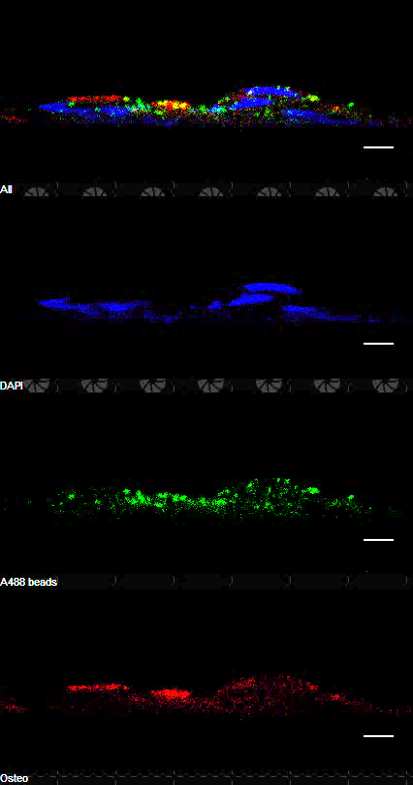

Supplement: second revision_Original image for microscopy studies.zip [file IDRD_A_2608235_SM4435.zip › Original Image for fig 003B_lower middle panel.tif]

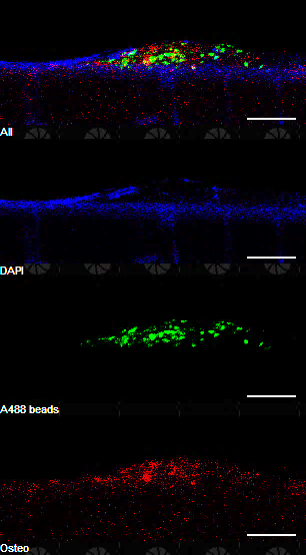

Supplement: second revision_Original image for microscopy studies.zip [file IDRD_A_2608235_SM4435.zip › Original Image for fig 003B_lower left panel.tif]

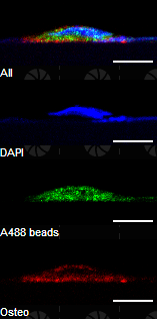

Supplement: second revision_Original image for microscopy studies.zip [file IDRD_A_2608235_SM4435.zip › Original Image for fig 003B_upper right panel.tif]

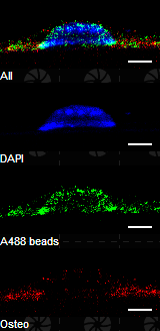

Supplement: second revision_Original image for microscopy studies.zip [file IDRD_A_2608235_SM4435.zip › Original Image for fig 003B_upper middle panel.tif]

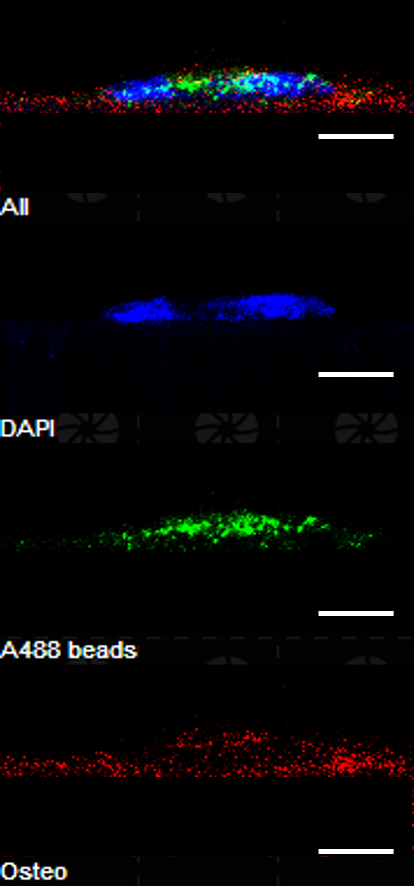

Supplement: second revision_Original image for microscopy studies.zip [file IDRD_A_2608235_SM4435.zip › Original Image for fig 003B_upper left panel.tif]

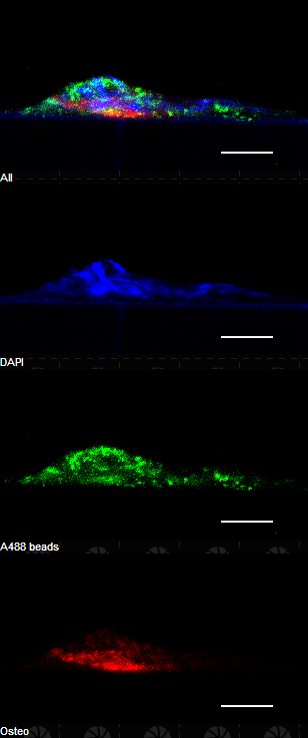

Supplement: second revision_Original image for microscopy studies.zip [file IDRD_A_2608235_SM4435.zip › Original Image for fig 003A_lower right panel.tif]

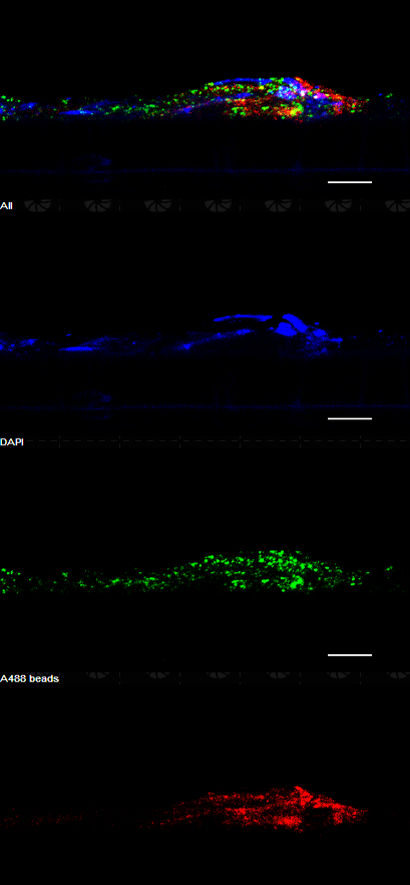

Supplement: second revision_Original image for microscopy studies.zip [file IDRD_A_2608235_SM4435.zip › Original Image for fig 003A_lower middle panel.tif]

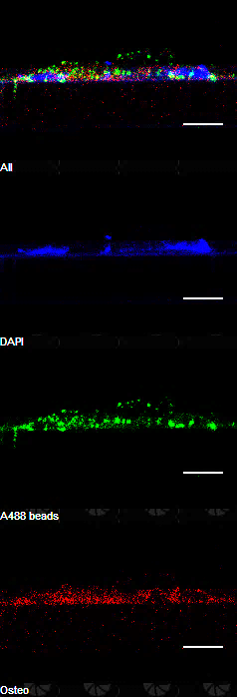

Supplement: second revision_Original image for microscopy studies.zip [file IDRD_A_2608235_SM4435.zip › Original Image for fig 003A_lower left panel.tif]

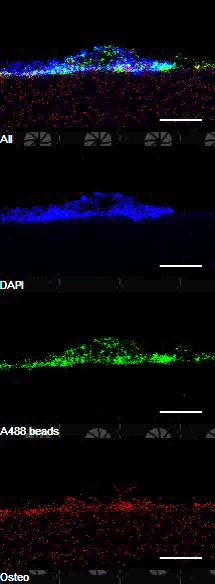

Supplement: second revision_Original image for microscopy studies.zip [file IDRD_A_2608235_SM4435.zip › Original Image for fig 003A_upper right panel.tif]

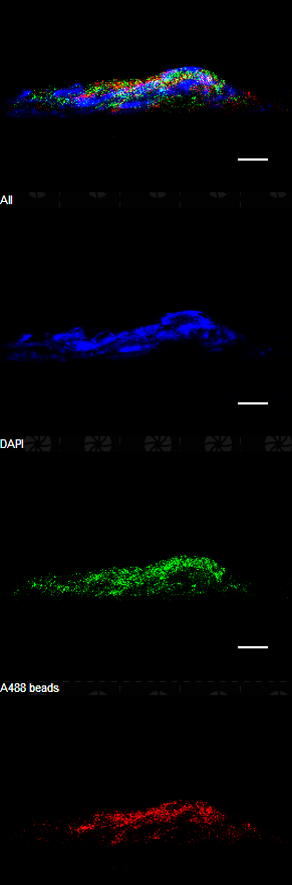

Supplement: second revision_Original image for microscopy studies.zip [file IDRD_A_2608235_SM4435.zip › Original Image for fig 003A_upper middle panel.tif]

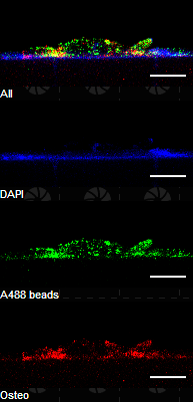

Supplement: second revision_Original image for microscopy studies.zip [file IDRD_A_2608235_SM4435.zip › Original Image for fig 003A_upper left panel.tif]

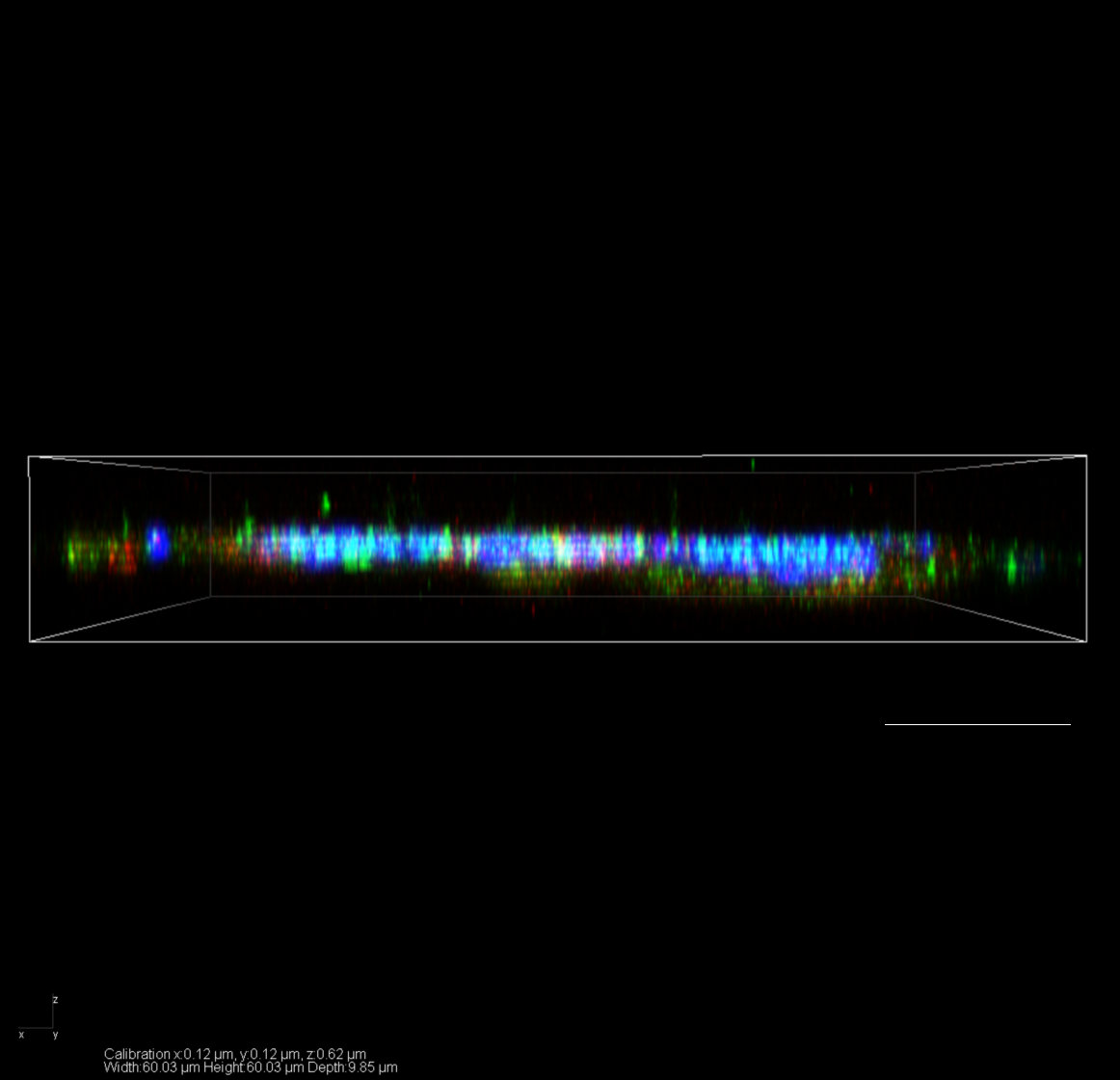

Supplement: second revision_Original image for microscopy studies.zip [file IDRD_A_2608235_SM4435.zip › Original Image for fig 002C.tif]

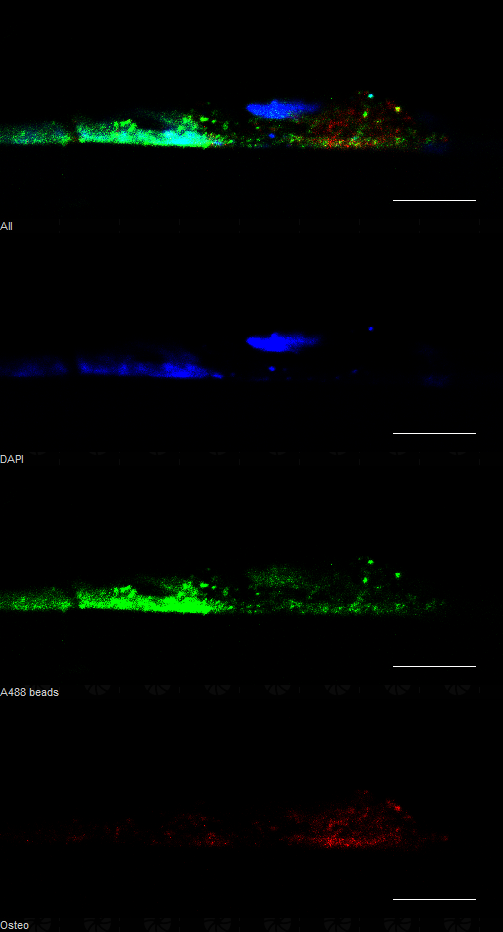

Supplement: second revision_Original image for microscopy studies.zip [file IDRD_A_2608235_SM4435.zip › Original Image for fig 002D.tif]

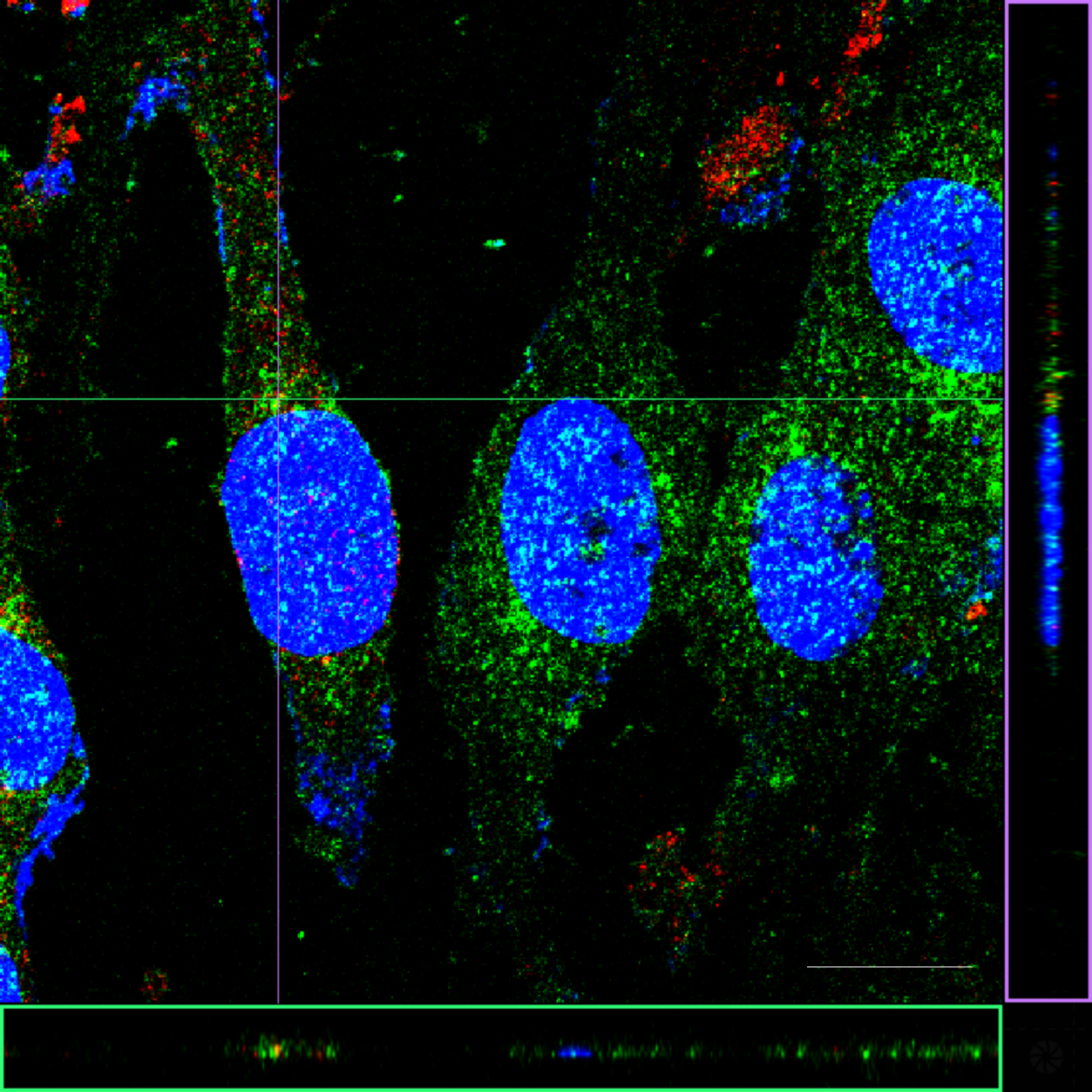

Supplement: second revision_Original image for microscopy studies.zip [file IDRD_A_2608235_SM4435.zip › Original Image for fig 002B.tif]

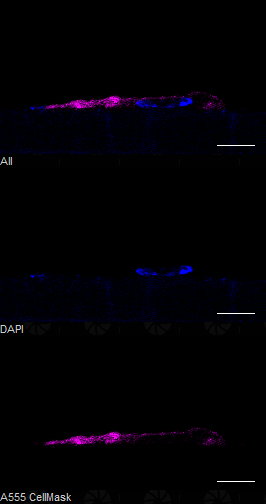

Supplement: second revision_Original image for microscopy studies.zip [file IDRD_A_2608235_SM4435.zip › Original Image for fig 001B_left panel.tif]

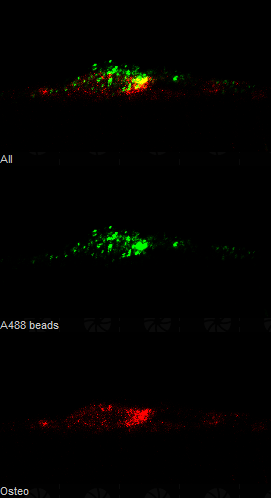

Supplement: second revision_Original image for microscopy studies.zip [file IDRD_A_2608235_SM4435.zip › Original Image for fig 005A_lower middle panel_green red merge.tif]

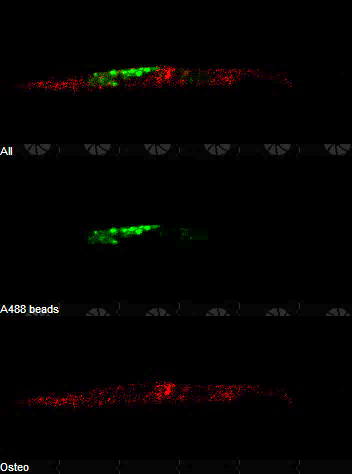

Supplement: second revision_Original image for microscopy studies.zip [file IDRD_A_2608235_SM4435.zip › Original Image for fig 006B_lower right panel_green red merge.tif]

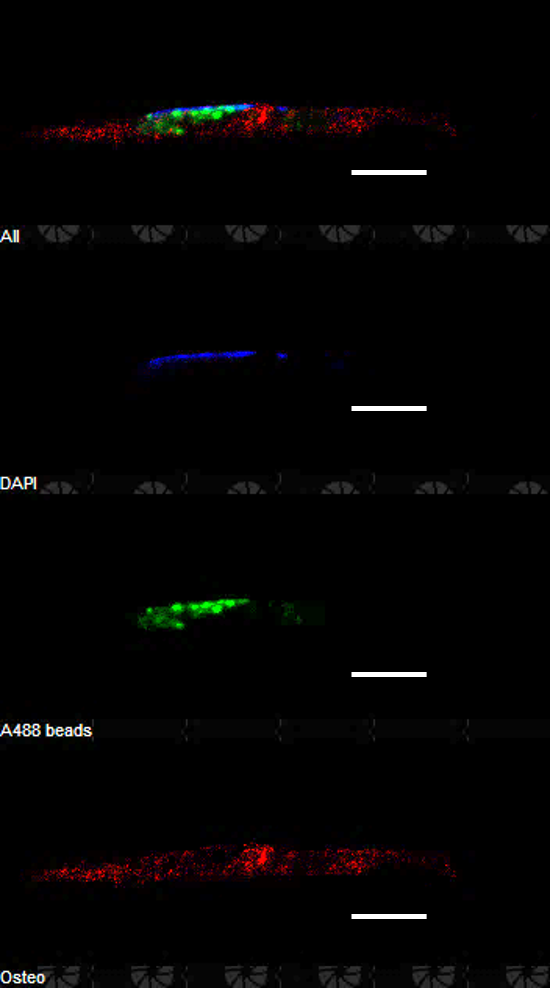

Supplement: second revision_Original image for microscopy studies.zip [file IDRD_A_2608235_SM4435.zip › Original Image for fig 006B_lower right panel.tif]

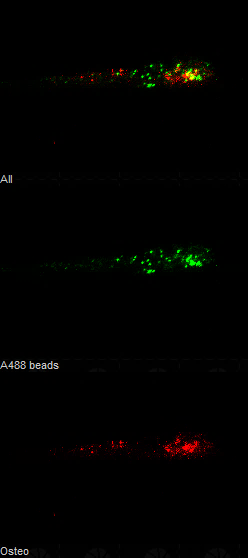

Supplement: second revision_Original image for microscopy studies.zip [file IDRD_A_2608235_SM4435.zip › Original Image for fig 005B_lower middle panel_green red merge.tif]

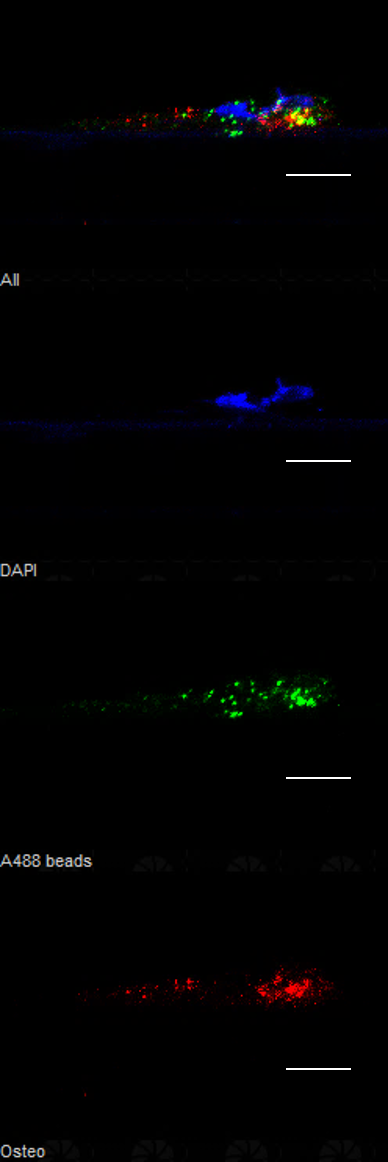

Supplement: second revision_Original image for microscopy studies.zip [file IDRD_A_2608235_SM4435.zip › Original Image for fig 005B_lower middle panel.tif]

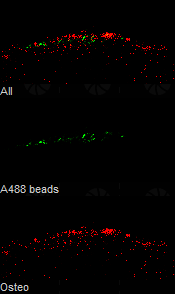

Supplement: second revision_Original image for microscopy studies.zip [file IDRD_A_2608235_SM4435.zip › Original Image for fig 005B_lower left panel_green red merge.tif]

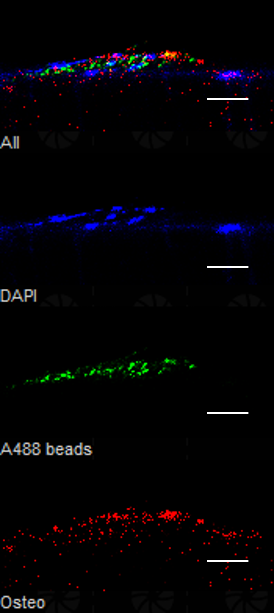

Supplement: second revision_Original image for microscopy studies.zip [file IDRD_A_2608235_SM4435.zip › Original Image for fig 005B_lower left panel.tif]
