# Supplementary material for: Cell swelling and upright mounting-based imaging for high-resolution visualization of intracellular trafficking across the BBB using conventional confocal microscopy
Source: Drug Deliv. 2026 Jan 6;33(1):2608235. doi: 10.1080/10717544.2025.2608235 (PMC12781939; doi:10.1080/10717544.2025.2608235)
Supplement: second_revision_20251216_supplementary_information Clean.docx [file IDRD_A_2608235_SM4434.docx]

**Supplementary information**

**Cell swelling and upright mounting-based imaging for high-resolution visualization of intracellular trafficking across the BBB using conventional confocal microscopy**

Da Hee Oh^1^, Ji Hee Kang^1^, O Hyun Lee^1^ and Young Tag Ko^1*^

**Affiliations:**

^1^College of Pharmacy and Gachon Institute of Pharmaceutical Sciences, Gachon University, Incheon, Republic of Korea 21936

*Corresponding author: Professor Young Tag Ko, College of Pharmacy, Gachon University, 191 Hambakmoe-ro, Yeonsu-gu, Incheon, Republic of Korea 21936

Tel.: 82-32-820-4923; Fax: 82-32-820-4829

E-mail address: [youngtakko@gachon.ac.kr](mailto:youngtakko@gachon.ac.kr)

Table S1. Apparent permeability coefficient (P_app_) values of A647-anti-TfR Ab across the *in vitro* BBB model at indicated time points, determined by fluorescence-based quantification. Values are expressed in cm/s × 10^-6^ and presented as mean ± SEM (n = 3).

| **(h)** | **P_app_ (cm/s × 10^-6^) ± SEM** |
| --- | --- |
| **1** | 0.749 ± 0.002 |
| **2** | 0.689 ± 0.002 |
| **4** | 0.574 ± 0.001 |

Table S2. Apparent permeability coefficient (P_app_) values of A647-anti-TfR Ab across the *in vitro* BBB model at indicated time points, determined by ELISA-based quantification. Values are expressed in cm/s × 10^-6^ and presented as mean ± SEM (n = 3).

| **(h)** | **P_app_ (cm/s × 10^-6^) ± SEM** |
| --- | --- |
| 1 | 0.757 ± 0.003 |
| 2 | 0.615 ± 0.003 |
| 4 | 0.588 ± 0.006 |

Table S3. Apparent permeability coefficient (P_app_) values of A647-Tf across the *in vitro* BBB model at indicated time points, determined by fluorescence-based quantification. Values are expressed in cm/s × 10^-6^ and presented as mean ± SEM (n = 3).

| **(h)** | **P_app_ (cm/s × 10^-6^) ± SEM** |
| --- | --- |
| 1 | 2.167 ± 0.003 |
| 2 | 2.218 ± 0.003 |
| 4 | 2.157 ± 0.002 |

Table S4. Apparent permeability coefficient (P_app_) values of A647-Tf across the *in vitro* BBB model at indicated time points, determined by ELISA-based quantification. Values are expressed in cm/s × 10^-6^ and presented as mean ± SEM (n = 3).

| **(h)** | **P_app_ (cm/s × 10^-6^) ± SEM** |
| --- | --- |
| 1 | 2.154 ± 0.002 |
| 2 | 2.221 ± 0.003 |
| 4 | 2.163 ± 0.002 |

**Table S5.** **Carrier × time interaction effects from two-way ANOVA of A647–Tf and A647–anti-TfR Ab co-localization**

| **Subcellular marker** | **p-value** |
| --- | --- |
| Rab5 | **** |
| EEA1 | ns |
| Rab7 | ** |
| VAMP7 | **** |
| LAMP1 | **** |
| Rab11 | * |
| VAMP3 | * |
| SNAP23 | ns |
| Syntaxin 4 | **** |

Interaction effects (carrier × time) were analyzed using two-way ANOVA followed by Tukey’s multiple comparisons test. Significant differences are indicated as p < 0.05 (*), < 0.01 (**), < 0.001 (***), < 0.0001 (****); ns = not significant.

Figure S1. Trans-endothelial electrical resistance (TEER) measurements before and after *in vitro* BBB permeability assay (n=3, mean ± SEM)

 Figure S2. Time-dependent stability of TEER in bEnd.3 monolayers under isotonic and hypotonic conditions. TEER values of bEnd.3 monolayers were measured for 10 min following exposure to 1X PBS or 0.5X PBS (n = 3). Dashed lines represent the baseline resistance of blank inserts measured under corresponding buffer conditions (58 Ω·cm² for 1X PBS and 128 Ω·cm² for 0.5X PBS). Mean TEER of bEnd.3 monolayers remained stable at ~307 Ω·cm² in 1X PBS and ~620 Ω·cm² in 0.5X PBS throughout the 10 min measurement.

**
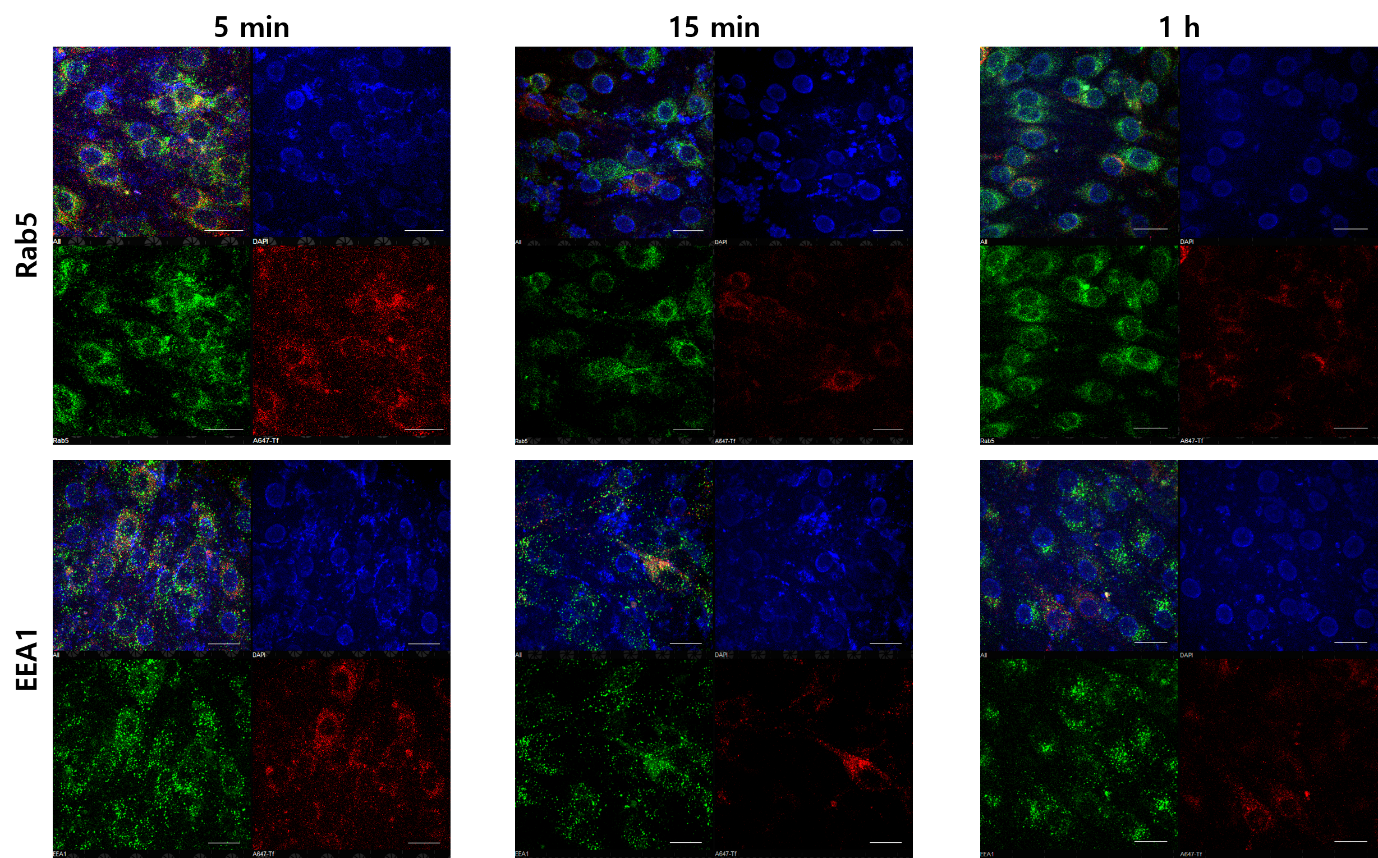
**

Figure S3. Confocal XY-plane images showing co-localization of A647-Tf with early endosome markers. Cells treated with A647-Tf (red) for 5 min, 15 min, or 1 h, then immunostained for early endosome markers Rab5 or EEA1 (green) and counterstained with DAPI (blue). Merged images (top left in each set) show co-localization between transferrin and endosomal markers. Scale bars: 25 µm.

**
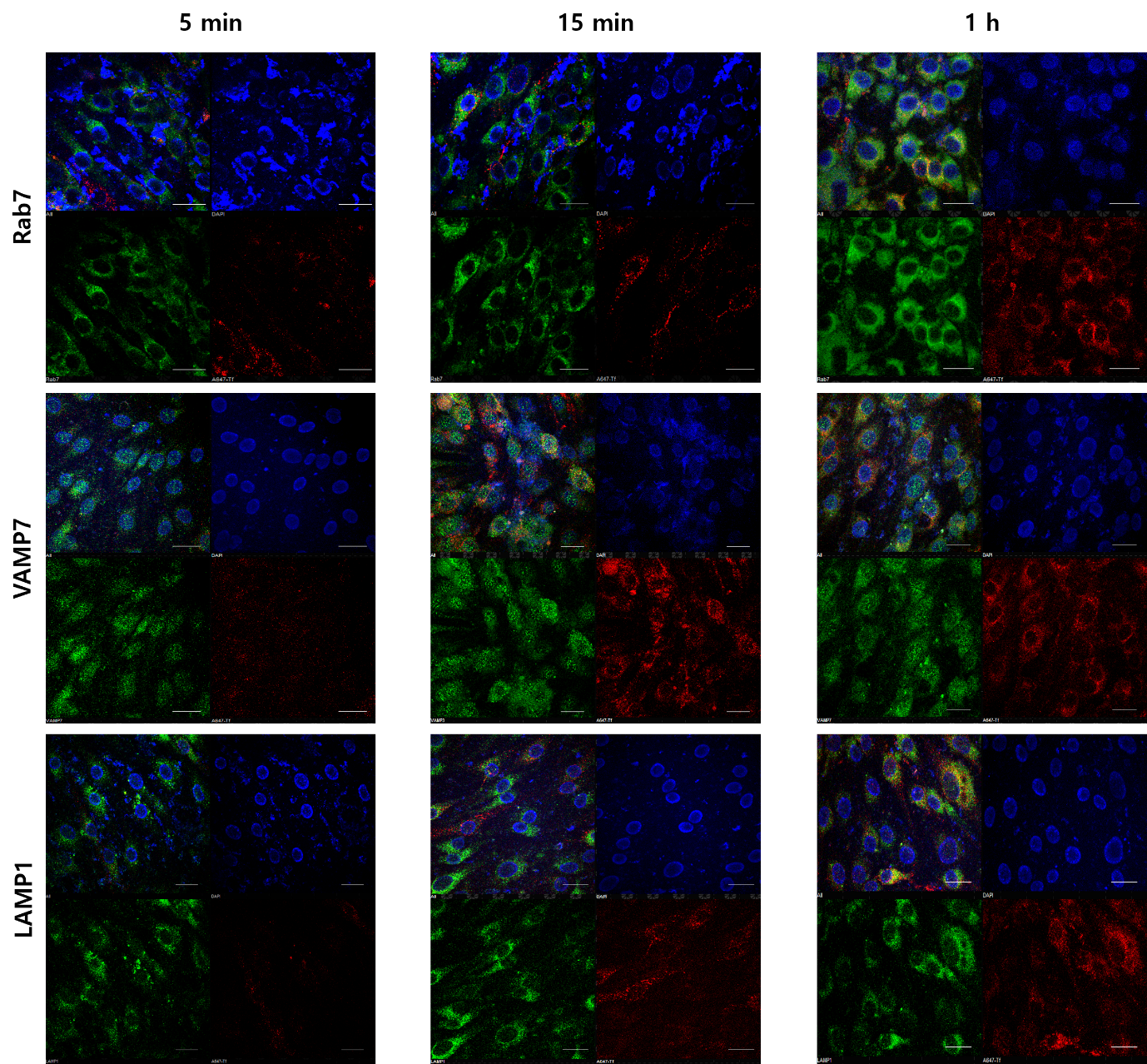
**

Figure S4. Confocal XY-plane images showing co-localization of A647-Tf with early Late endosome/Lysosome markers. Cells treated with A647-Tf (red) for 5 min, 15 min, or 1 h, then immunestained for Late endosome/Lysosome markers Rab7, VAMP7 or LAMP1 (green) and counterstained with DAPI (blue). Merged images (top left in each set) show co-localization between transferrin and endosomal markers. Scale bars: 25 µm.

**
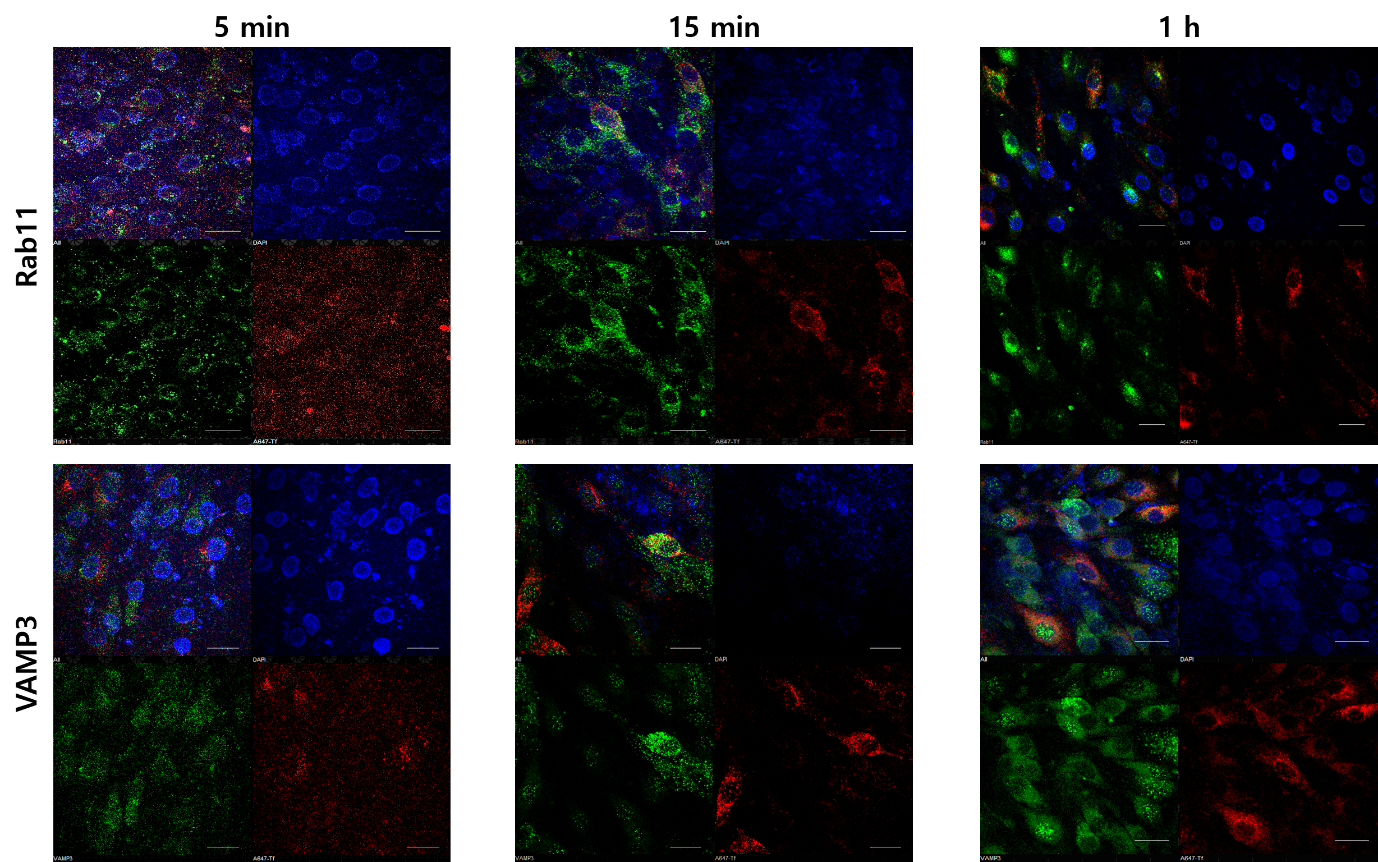
**

Figure S5. Confocal XY-plane images showing co-localization of A647-Tf with recycling endosome markers. Cells treated with A647-Tf (red) for 5 min, 15 min, or 1 h, then immunostained for recycling endosome markers Rab11 or VAMP3 (green) and counterstained with DAPI (blue). Merged images (top left in each set) show co-localization between transferrin and endosomal markers. Scale bars: 25 µm.

**
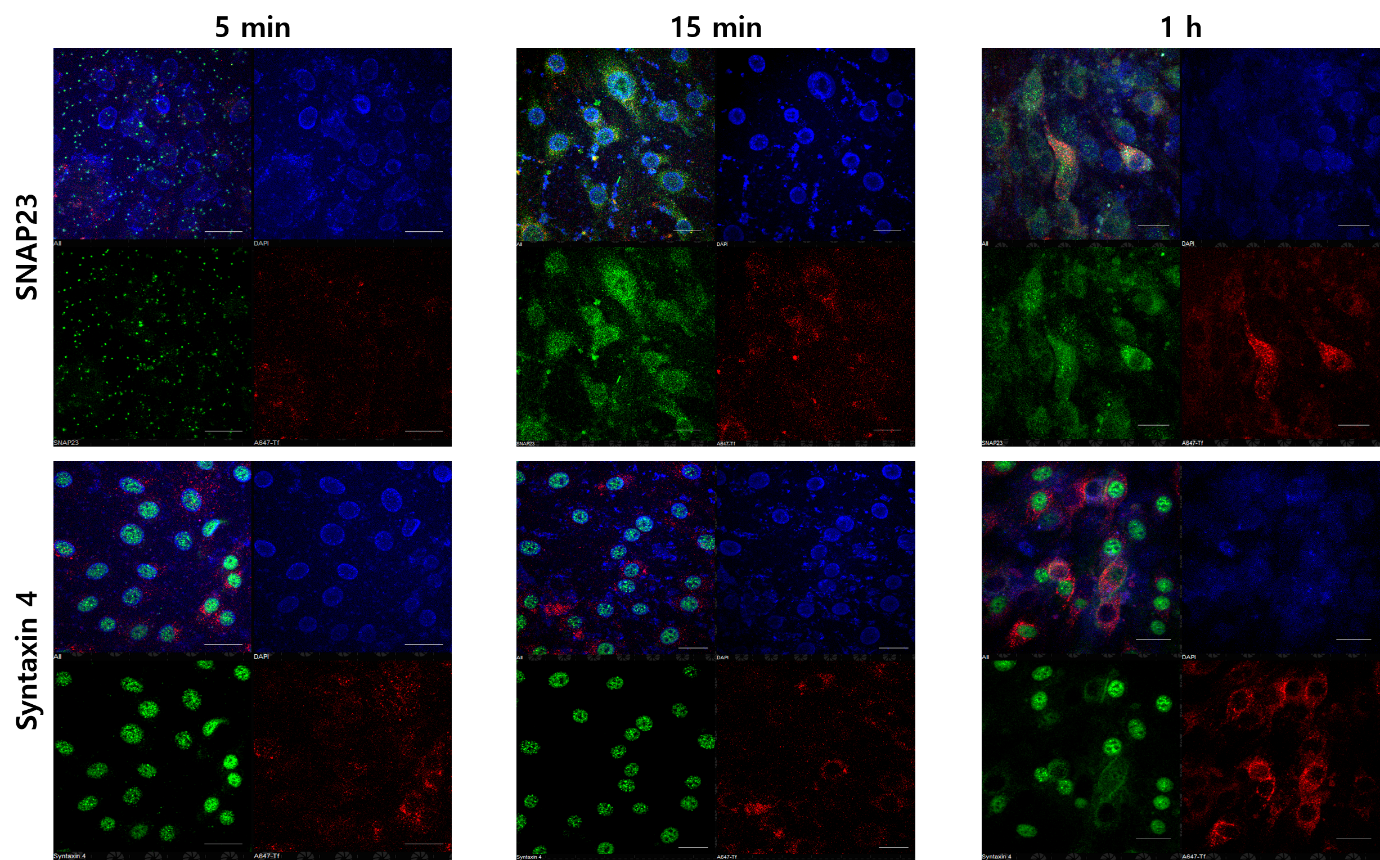
**

Figure S6. Confocal XY-plane images showing co-localization of A647-Tf with basolateral membrane markers. Cells treated with A647-Tf (red) for 5 min, 15 min, or 1 h, then immunostained for basolateral membrane markers SNAP23 or Syntaxin 4 (green) and counterstained with DAPI (blue). Merged images (top left in each set) show co-localization between transferrin and endosomal markers. Scale bars: 25 µm.

**
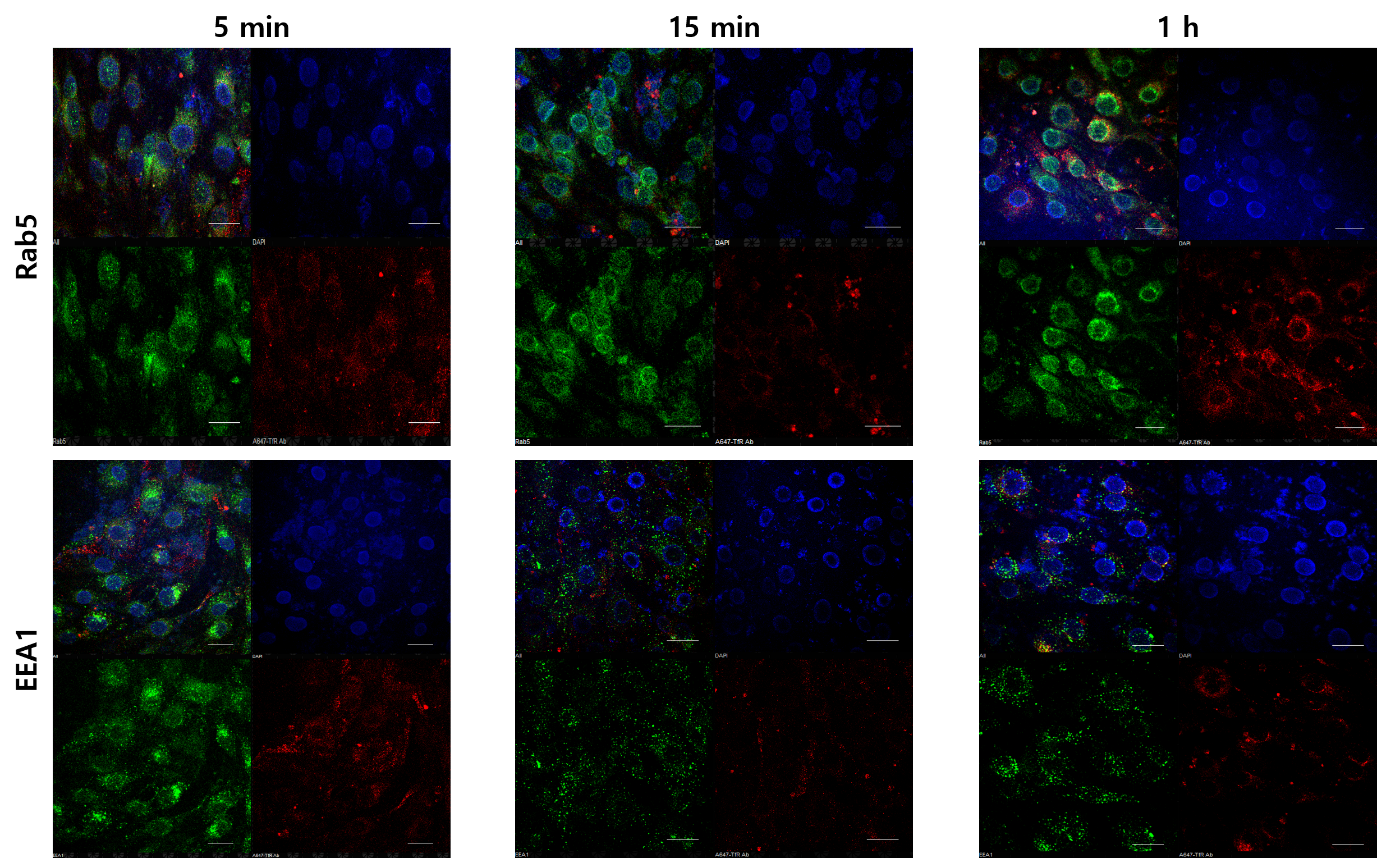
**

Figure S7. Confocal XY-plane images showing co-localization of A647-anti-TfR Ab with early endosome markers. Cells treated with A647-anti-TfR Ab (red) for 5 min, 15 min, or 1 h, then immunostained for early endosome markers Rab5 or EEA1 (green) and counterstained with DAPI (blue). Merged images (top left in each set) show co-localization between transferrin and endosomal markers. Scale bars: 25 µm.

**
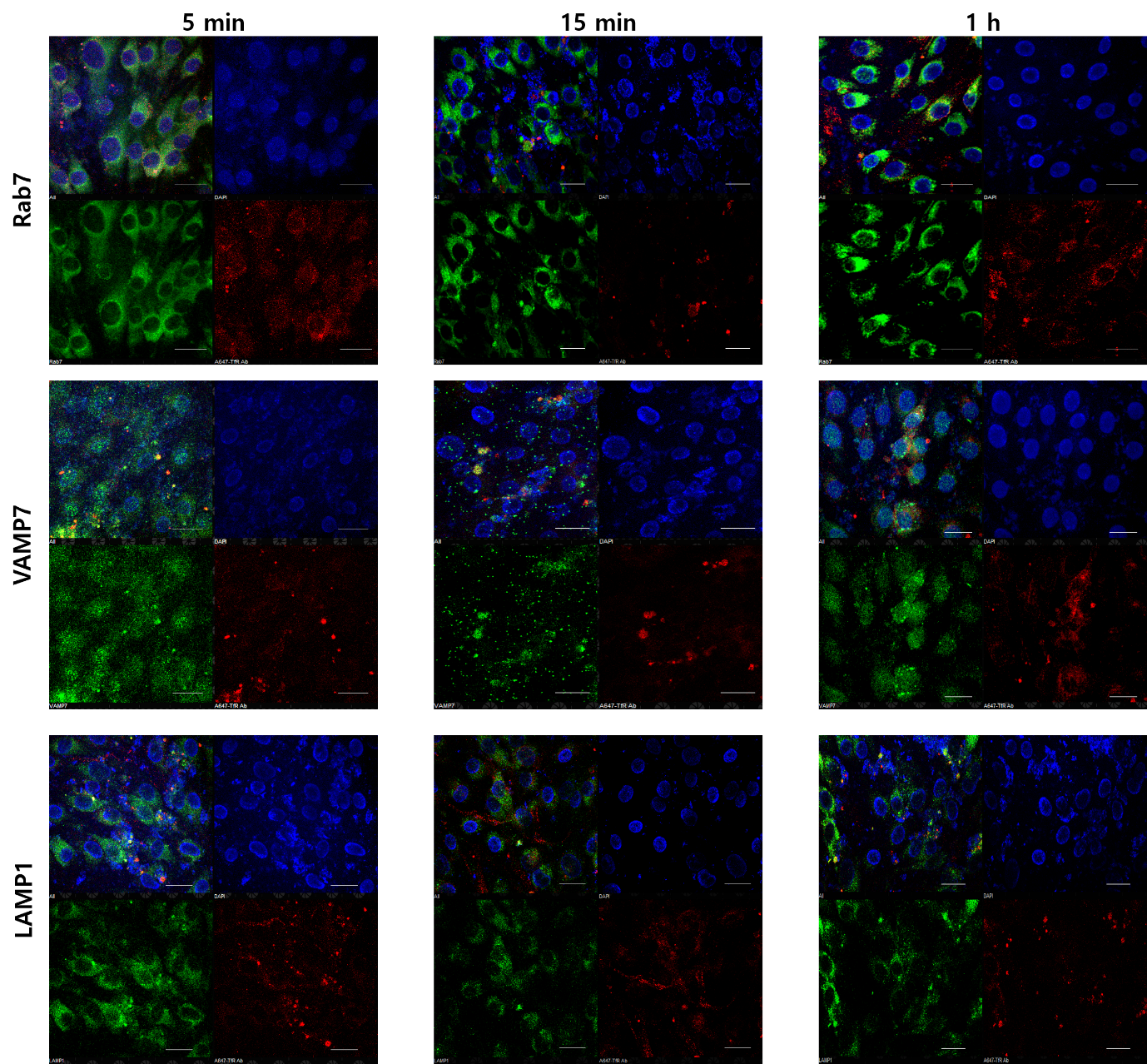
**

Figure S8. Confocal XY-plane images showing co-localization of A647-anti-TfR Ab with early Late endosome/Lysosome markers. Cells treated with A647-anti-TfR Ab (red) for 5 min, 15 min, or 1 h, then immunostained for Late endosome/Lysosome markers Rab7, VAMP7 or LAMP1 (green) and counterstained with DAPI (blue). Merged images (top left in each set) show co-localization between transferrin and endosomal markers. Scale bars: 25 µm.

**
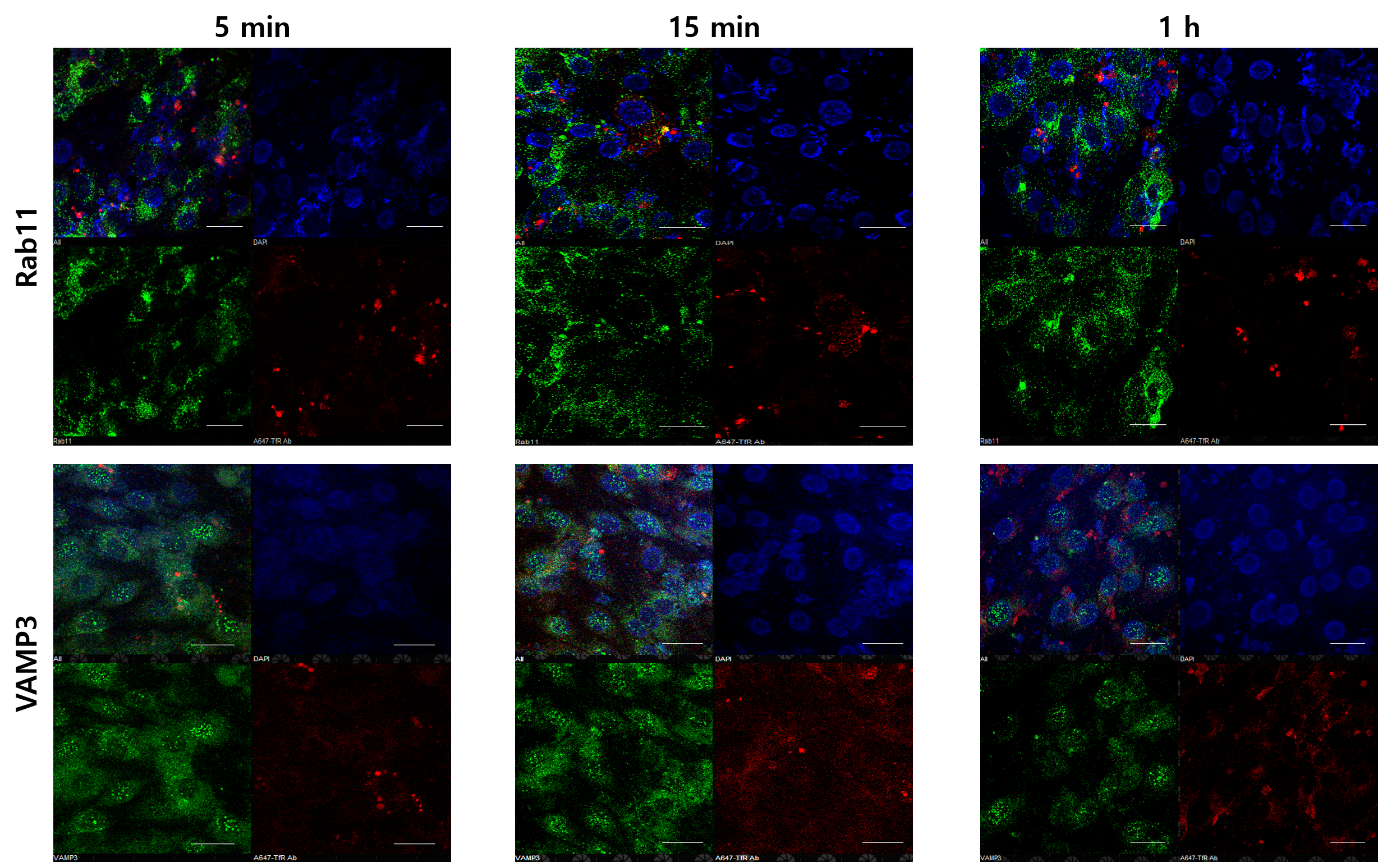
**

Figure S9. Confocal XY-plane images showing co-localization of A647-anti-TfR Ab with recycling endosome markers. Cells treated with A647-anti-TfR Ab (red) for 5 min, 15 min, or 1 h, then immunostained for recycling endosome markers Rab11 or VAMP3 (green) and counterstained with DAPI (blue). Merged images (top left in each set) show co-localization between transferrin and endosomal markers. Scale bars: 25 µm.

**
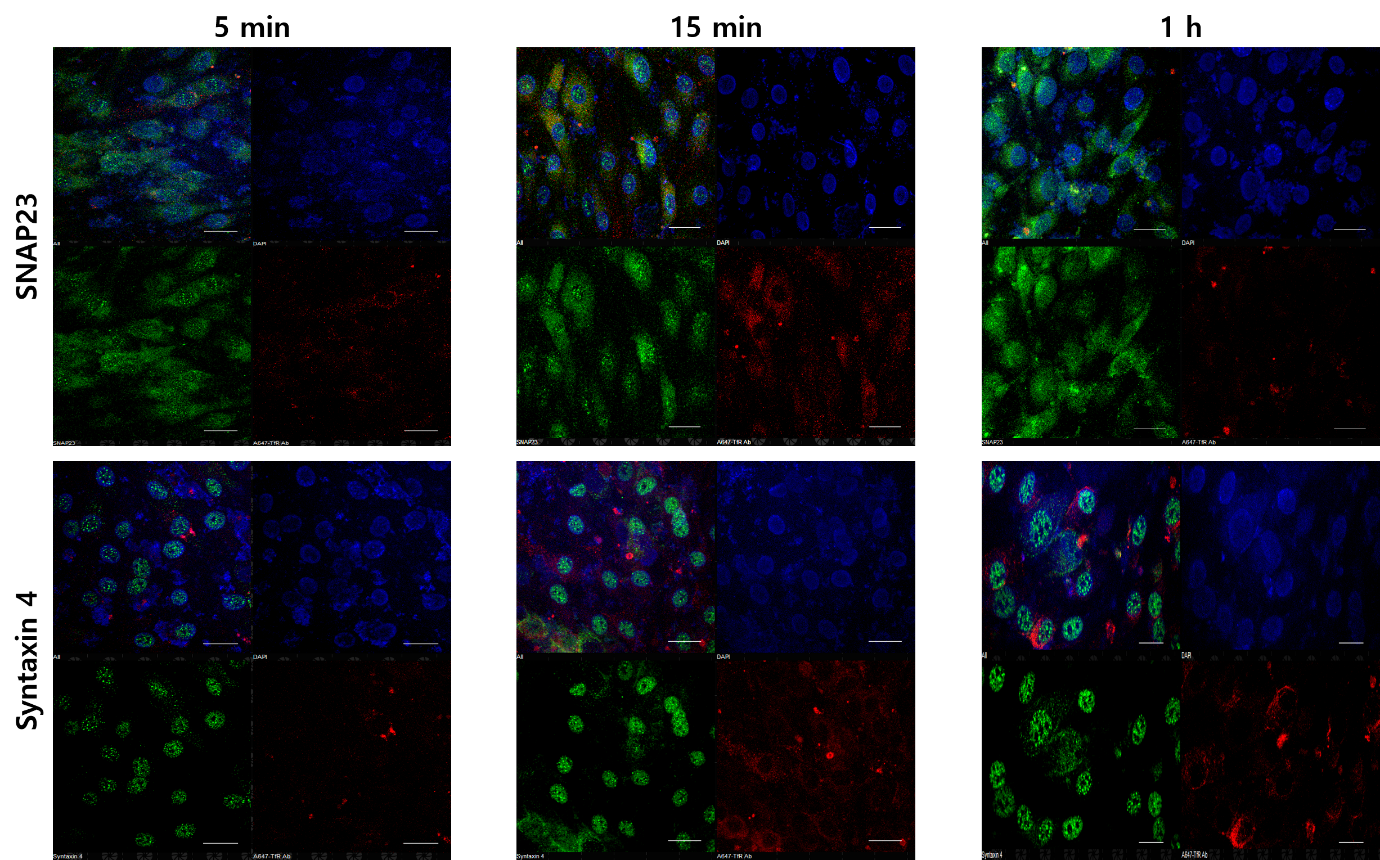
**

Figure S10. Confocal XY-plane images showing co-localization of A647-anti-TfR Ab with basolateral membrane markers. Cells treated with A647-anti-TfR Ab (red) for 5 min, 15 min, or 1 h, then immunostained for basolateral membrane markers SNAP23 or Syntaxin 4 (green) and counterstained with DAPI (blue). Merged images (top left in each set) show co-localization between transferrin and endosomal markers. Scale bars: 25 µm.
